# The Relationship Between Austrian Tax Auditors and Self-Employed Taxpayers: Evidence From a Qualitative Study

**DOI:** 10.3389/fpsyg.2019.01034

**Published:** 2019-05-13

**Authors:** Katharina Gangl, Barbara Hartl, Eva Hofmann, Erich Kirchler

**Affiliations:** ^1^Department of Economic and Social Psychology, University of Göttingen, Göttingen, Germany; ^2^Competence Center for Empirical Research Methods, Vienna University of Economics and Business, Vienna, Austria; ^3^Danube University Krems, Krems, Austria; ^4^Department of Applied Psychology, Work, Education and Economy, University of Vienna, Vienna, Austria

**Keywords:** public institutions, public administration, tax compliance, tax evasion, cooperation, power, trust

## Abstract

A constructive, highly professional relationship between tax authorities and taxpayers is essential for tax compliance. The aim of the present paper was to explore systematically the determinants of this relationship and related tax compliance behaviors based on the extended slippery slope framework. We used in-depth qualitative interviews with 33 self-employed taxpayers and 30 tax auditors. Interviewees described the relationship along the extended slippery slope framework concepts of power and trust. However, also novel sub-categories of power (e.g., setting deadlines) and trust (e.g., personal assistance) were mentioned. Furthermore, also little-studied categories of tax behavior emerged, such as accepting tax behavior, e.g., being available to the tax authorities, or stalling tax behavior, e.g., the intentional creation of complexity. The results comprehensively summarize the determinants of the tax relationship and tax compliance behaviors. Additionally, results highlight future research topics and provide insights for policy strategies.

## Introduction

The quality of the interaction climate between tax authorities and taxpayers is increasingly deemed important for tax compliance. Whereas studies historically considered simple stimulus-response mechanisms such as the influence of an increased tax rate on tax compliance (Allingham and Sandmo, 1972), today practice, and research increasingly considers the relationship between tax authorities and taxpayers (Braithwaite, 2002; Feld and Frey, 2002; Kirchler, 2007). For instance, the Organization for Economic Co-operation and Development (Organisation for Economic Co-operation and Development [OECD], 2013) promotes cooperative relationships between tax authorities and taxpayers, and encourages tax administrations to build up trust-based interactions with honest taxpayers. These new policy perspectives are supported by theories such as the responsive regulation theory (Braithwaite, 2002, 2003) and the slippery slope framework of tax compliance (Kirchler, 2007; Kirchler et al., 2008). Responsive regulation theory proposes that tax authorities manage their relationships with taxpayers as response to the taxpayers’ motivational postures (Braithwaite, 2007). Akin to other theoretical accounts (e.g., Feld and Frey, 2007; Luttmer and Singhal, 2014), the slippery slope framework suggests that two key determinants (i.e., the power of authorities and trust in authorities) related to classical economic deterrence factors such as audits and fines, as well as psychological factors related to fairness and reciprocity rules, shape the tax climate, and in turn, tax compliance (Gangl et al., 2015). However, in spite of practical acknowledgment and the existence of several theoretical models concerning the relationship climate, empirical knowledge regarding the determinants that shape the interaction process and resulting tax behavior remains to be further investigated.

The first aim of the present explorative research is, based on in-depth interviews with Austrian taxpayers and tax auditors, to empirically examine the theoretical constructs regarding the actors’ relationship and tax compliance behaviors that are defined in the extended slippery slope framework. The second aim is, to identify novel sub-categories about tax behavior and its perceived relationship antecedents to generate new research hypotheses. In contrast to quantitative experimental and survey approaches, which often over-simplify compliance behavior, use closed questions and investigate student samples, our interview-based approach which includes both tax auditors and self employed taxpayers, will increase the practical relevance of insights into the tax compliance relationship, allowing to detect concepts that have not been considered so far. There is only one previous study, to the best of our knowledge, which investigates taxpayers and tax auditors (Kirchler et al., 2006).

## Theoretical Background

### The Extended Slippery Slope Framework

The slippery slope framework was developed as a conceptual tool to organize research on tax compliance determinants into two dimensions: power of authorities and trust in authorities (Kirchler et al., 2008). Power defines research predominantly in economics and is defined as the potential and ability of an entity to influence another entity’s behavior (Freiberg, 2010). Trust summarizes predominantly psychological approaches and refers to taxpayers’ perception of tax authorities’ competence and their behavior in the interest of the community (Kirchler et al., 2008). Both determinants affect tax behavior, although power fosters an antagonistic relationship climate, whereas trust leads to a synergistic climate. In the case of the former, compliance is based on strict enforcement, whereas in the latter, compliance is based on voluntary or committed cooperation (Kirchler et al., 2008).

According to the extended slippery slope framework (Gangl et al., 2015), power, and trust are further differentiated. For instance, the power of authorities is divided into coercive and legitimate power (Turner, 2005; Tyler, 2006). Coercive power is related to classical economic theories on tax compliance, incentivizing a rational taxpayer through punishment, and rewards (Becker, 1968; Allingham and Sandmo, 1972). Legitimate power, on the other hand, summarizes social and legal psychological ideas about influencing citizens by gaining their acceptance through being perceived inter alia as legitimized and as acting professionally (Tyler, 2006). The dimension of trust in authorities refers to dual process theories (i.e., system 1 and system 2) on cognition (e.g., Kahneman, 2003) and trust (e.g., Lewicki and Bunker, 1996; Misztal, 1996), and differentiates trust in reason-based and implicit trust (Castelfranchi and Falcone, 2010). Reason-based trust is defined as taxpayers’ deliberate conclusion that the tax authorities can be trusted based on a series of criteria such as tax authorities’ perceived goals. Finally, implicit trust is defined as inter alia taxpayers’ automatic and unconscious trust reactions to stimuli such as an official-looking document (Gangl et al., 2015). In order to explain the development of and change in tax relationships, the extended slippery slope framework integrates a wide range of theories on cognition (Kahneman, 2003; Evans, 2008), leadership (French and Raven, 1959; Avolio and Bass, 1991), and social and organizational relationships (Lewin et al., 1939; Ouchi, 1979; Haslam and Fiske, 1999; Adler, 2001; Alm and Torgler, 2011).

#### Tax Authorities’ Power

In the following section, and based on the extended slippery slope framework’s concepts of power and trust, we review and categorize research on tax compliance determinants, particularly relationship determinants as well as the different qualities of tax compliance behaviors, by incorporating the latest findings from psychological, economic, legal, and administrative research. After each section, we present our research questions.

##### Coercive power

Coercive power is defined as the possibility of deterring tax evasion and fostering tax honesty by using enforcement and incentives. Based on the theory of the social bases of power (French and Raven, 1959; Raven, 1993; Raven et al., 1998) and in line with research on taxes, we differentiate between punishment power and reward power as forms of coercive power. Although Raven et al. (1998) called this forms of power harsh power, we stay with the term coercive power as previous research on regulation of citizens’ behavior (Turner, 2005).

Punishment power in the form of audits and fines is probably the instrument that is most commonly used by tax authorities to enforce compliance (e.g., Andreoni et al., 1998; Kastlunger et al., 2009; Kirchler et al., 2010; Castro and Scartascini, 2013). Punishment power operates in line with the standard economic model of criminal behavior (Becker, 1968) and the standard model of tax compliance (Allingham and Sandmo, 1972). The models state that individuals evaluate the probability and consequences of audits and punishment of law violation. Given that individuals and firms are assumed to seek profit maximization, next to income and tax rate, compliance is essentially considered a function of detection probability, and severity of sanctions (Allingham and Sandmo, 1972; Srinivasan, 1973). Thus, all possibilities to coercively enforce compliance by maximizing detection, e.g., based on third-party information or withholding taxes, increase tax honesty (Luttmer and Singhal, 2014). More recent research has additionally examined public disclosure in the form of transparent tax returns (Bø et al., 2014) or black lists (Perez-Truglia and Troiano, 2015) as coercive measures to deter tax evasion.

*Research question 1a:* Do tax auditors and taxpayers perceive categories of punishment power (audits, fines and public disclosure)?

*Research question 1b:* Do tax auditors and taxpayers perceive categories of punishment that have not been considered in the literature?

Akin to punishment power as negative reinforcement, reward power in the form of positive reinforcement is considered in the decision to comply or not comply (Feld et al., 2006). Rewards for compliant behavior are also considered a form of coercion, and are likely to crowed-out intrinsic motivation to cooperate (Deci, 1971; Frey, 1997). Tax research on the effects of monetary and non-monetary rewards is rather scarce and findings are inconsistent (Falkinger and Walther, 1991; Torgler, 2002; Feld et al., 2006; Feld and Frey, 2007; Kastlunger et al., 2010). For instance, studies examine the impact of lottery tickets (Bazart and Pickhard, 2011), wellness vouchers (Koessler et al., 2016), or the promise of privileged treatment (Simone et al., 2013). Although, rare empirical evidence for the effect exists, some non-governmental organizations (NGOs) hope to increase tax compliance by honoring honest taxpayers through positive disclosure (see for example^1^).

*Research question 2a:* Do tax auditors and taxpayers perceive categories of reward power (monetary, non-monetary or positive disclosure)?

*Research question 2b:* Do tax auditors and taxpayers perceive categories of reward power that have not been considered in the literature?

##### Legitimate power

In the extended slippery slope framework, legitimate power is characterized by the legitimacy of the tax authority (Hofmann et al., 2014; Gangl et al., 2015). Based on theories of legitimacy (Tyler, 1990, 2006; Turner, 2005) and social bases of power (French and Raven, 1959; Raven et al., 1998), legitimate power is seen as soft power, comprising positional power, information power, expert power, and referent power. Although Raven et al. (1998) called this form of power soft power, we stick with the terminology of the regulation of citizens’ behavior, and call soft power legitimate power (Turner, 2005). Positional power refers to the perception that the authority has the legal right to levy taxes. Information power is based on tax authorities’ circulation of relevant information. Expert power means that tax authorities are perceived as skilled and professionally trained. Referent power refers to authorities’ capacity to influence taxpayers based on their own positive image (Raven et al., 1998). The different categories of legitimate power are related to the perception of a transparent and fair tax system (Wenzel, 2002; Bradford et al., 2014), facilitating the taxpayer’s voice, and participation (Pommerehne and Weck-Hannemann, 1996; Feld and Tyran, 2002) in levying and spending taxes. Rich empirical data regarding the effectiveness of proxies for legitimate power indicate a positive relationship between customer orientation, perceived legitimacy, provision of relevant information and supportive procedures, knowledge and skills, and tax compliance (Alm et al., 2010; Hartner et al., 2011; Gangl et al., 2013; Hofmann et al., 2014).

*Research question 3a:* Do tax auditors and taxpayers perceive categories of legitimate power (positional, expert, information and referent)?

*Research question 3b:* Do tax auditors and taxpayers perceive forms of legitimate power that have not been considered in the literature?

#### Taxpayers’ Trust in the Tax Authority

Citizens’ trust in authorities is of paramount importance with regard to law compliance (e.g., Tyler, 1997; Jackson et al., 2012) and this is particularly relevant in the tax relationship (e.g., Scholz and Lubell, 1998; Feld and Frey, 2002; Murphy, 2004; Kirchler et al., 2008; Hammar et al., 2009; van Dijke and Verboon, 2010). Taxpayers trust the tax authority either deliberately or implicitly (Castelfranchi and Falcone, 2010; Gangl et al., 2015).

##### Implicit trust

Implicit trust in the tax authority originates from an automatic, unconscious reaction based on associative and conditional learning processes (Castelfranchi and Falcone, 2010). Based on such associative experiences, individuals learn that in some situations they can trust to a greater degree than in others. For instance, if taxpayers possess positive experiences of interacting with the tax authority, they are more likely to trust them in the future without thinking about it (Gangl et al., 2015; see also trust based on reciprocity and reputation, e.g., King-Casas et al., 2005). Implicit trust is related to a perceived shared identity and shared values (Castelfranchi and Falcone, 2010). Individuals are more likely to trust those who they perceive similar to themselves (e.g., concerning sociodemographic background) and who share their views, interests and values (Kirchler et al., 2006). Relatedly, a perception that the tax authorities treat taxpayers as equal partners and exhibit empathy for their problems is likely to trigger implicit trust (Gangl et al., 2015). Finally, cues such as official documents, smiling faces and friendly voices can also stimulate automatic trust. Empirical studies show that proxies of implicit trust such as nudges (Behavioural Insights Team, 2011; Chirico et al., 2017) of social norms (Hallsworth et al., 2017) or reminders of a shared national identity (Gangl et al., 2016a,b) can increase tax compliance. However, the quantitative approach of most preexisting studies may explain why empirical evidence regarding the relevance of implicit trust on tax behavior remains scarce (Hofmann et al., 2014). Nevertheless, many tax authorities seek to trigger implicit trust, such as through advertisements or appealing website designs.

*Research question 4a:* Do tax auditors and taxpayers perceive categories of implicit trust (automatic trust, experience, shared values, empathy, and perception of being equal stakeholders)?

*Research question 4b:* Do tax auditors and taxpayers perceive categories of implicit trust that have not been considered in the literature?

##### Reason-based trust

Reason-based trust is based on deliberate considerations concerning taxpayers’ dependency on the tax authorities and the importance of tax authorities’ goals (Castelfranchi and Falcone, 2010; Gangl et al., 2015). In addition, taxpayers consider internal factors of the tax authorities such as competence, motivation, and benevolence and external factors, which may be relevant for the work of the authorities, such as economic and political conditions (Castelfranchi and Falcone, 2010; Hofmann et al., 2014; Gangl et al., 2015). Considerable empirical and theoretical evidence exists concerning the positive effects of deliberate forms of trust on cooperation, such as knowledge-based trust (Lewicki and Bunker, 1996) and rational trust (Ripperger, 1998). Conceptually, reason-based trust, especially internal factors, overlap with legitimate power (e.g., Malhotra and Murnighan, 2002; Hofmann et al., 2017a). They represent two sides of the same tax relationship: the legitimate power of the authorities is a perception of influence, and reason-based trust of taxpayers is the decision to be vulnerable based on the influencing entity and its environment. Numerous studies have demonstrated that reason-based trust (e.g., Murphy, 2004; Wahl et al., 2010; Gangl et al., 2013; Kogler et al., 2013) and its proxies such as perceived institutional quality and corruption (e.g., Cummings et al., 2009; Torgler and Schneider, 2009; Gangl et al., 2017) are essential for tax compliance.

*Research question 5a:* Do tax auditors and taxpayers perceive categories of reason-based trust (dependency, shared goals, internal factors and external factors)?

*Research question 5b:* Do tax auditors and taxpayers perceive categories of reason-based trust that have not been considered in the literature?

#### Tax Compliance Behavior

In the slippery slope framework, and similar to numerous theoretical and empirical accounts, only small subsets of tax compliance behaviors (such as honest payment and tax avoidance; see however Kirchler and Wahl, 2010, who developed scales on voluntary compliance, enforced compliance, tax avoidance, and evasion) are distinguished (Kirchler et al., 2008; Gangl et al., 2015). Furthermore, in the seminal work by Allingham and Sandmo (1972) and Srinivasan (1973), tax compliance is defined as the amount of honestly paid, or evaded tax. Most empirical work applying laboratory experiments and surveys is based on this simplified view on tax compliance (Alm et al., 1995; Hartl et al., 2015; Hofmann et al., 2017b). However, practitioners such as tax administrations (Organisation for Economic Co-operation and Development [OECD], 2004) hold a more complex understanding of tax compliance. They see it as consisting of, e.g., correct registration as a taxpayer, completing tax reports on time, reporting complete and accurate information, and paying taxes on time. Others differentiate between filing compliance, payment compliance, and reporting compliance (Brown and Mazur, 2003), or administrative compliance (i.e., registering, reporting, and time requirements) and technical compliance (i.e., taxes are calculated based on the technical requirements of the law, Organisation for Economic Co-operation and Development [OECD], 2001). Finally, tax authorities distinguish commercial tax avoidance as legal tax reduction within the brackets of the law (e.g., claiming refund for investments) from aggressive tax avoidance, as tax reduction against the spirit of the law (e.g., cross-border profit shifting and tax flight; Organisation for Economic Co-operation and Development [OECD], 2001).

*Research question 6a:* Do tax auditors and taxpayers perceive different categories of tax compliance behavior (honest taxpaying, tax evasion, tax registration, timely filing, correct reporting, commercial tax planning, and aggressive tax planning)?

*Research question 6b:* Do tax auditors and taxpayers perceive categories of tax behaviors that have not been considered in the literature?

## Method: Qualitative Interview Study

In order to answer the research questions and to depict the perception of the tax stakeholders, we followed a qualitative approach and conducted semi-structured interviews. Qualitative psychological research investigates the distinctive characteristics of experience of persons and is usually distinguished from quantitative methods adapted from natural sciences (Fischer, 2005). A qualitative approach is appropriate for investigating exploratory questions, such as the tax auditors’ and taxpayers’ perception of power and trust, as well as tax behaviors, as it gives voice to the subjective experience of the interviewees. Our qualitative approach builds on social constructivism and social representations theory, proposing that knowledge and attitudes about tax issues are gained through social interaction, communication and discussion in peer-groups, and insights from media reports (c.f., Moscovici, 1998; Peters, 2010).

We interviewed self-employed taxpayers and tax auditors as relevant stakeholders. We choose self-employed because compared to employed taxpayers in Austria their taxes are not withheld by the employer; rather they need to declare their gross income and pay taxes out of pocket. Thus, they have likely more experience with interactions with the tax authorities. In the following section, we present the sample, recruitment technique and interview procedures for both self-employed taxpayers and auditors. The results are presented subsequently.

### Self-Employed Taxpayers

#### Sample

In total, 33 Austrian self-employed taxpayers with small to medium size businesses participated in the study. Participants (15 of whom were female) were on average 44.34 years old (*N* = 32; one person did not indicate his/her age; *SD*_age_ = 11.69), and had on average 10.58 years (*SD* = 10.31) of experience as self-employed persons. The number of employees working for the self-employed taxpayers ranged from 0 (48.5%) to 50 (5.9%); 41.2% of those who employed personnel claimed to only have one employee. The majority of participants reported an annual turnover of less than 25,000 EUR (nine taxpayers), or between 25,000 EUR and 50,000 EUR (nine taxpayers). The majority of self-employed taxpayers (17 taxpayers) utilized a tax advisor. Concerning their experience with the tax authority, 11 taxpayers reported that they had been audited at least once.

#### Procedure and Material

Interviewees were recruited via a market research agency in 2013. All interviews were conducted by one interviewer, accompanied by two assistants, who tape-recorded the interviews. Interviews were semi-structured, lasting between 30 and 90 min. Following the interview, a short questionnaire was completed to gather information regarding the participants’ demographics and their businesses (see Supplementary Material). Interviews opened with a general question concerning taxpayers’ experience with the tax authority, thus affording them full freedom of expression. Subsequent questions delved into the tax authorities’ potential to affect tax behavior (power of the tax authority), taxpayers’ trust in the authority, and the impact of power and trust on their tax compliance. The interview questions were on taxes in general and did not specify a specific kind of tax. The interview guideline and questions were developed with the help of an experienced advisory board of tax researchers, including improvement loops based on test interviews. The interview materials can be found in the Supplementary Material, the transcripts can be found at osf.io/nv285/. Participants were remunerated with 50 EUR (approximately 53.35 USD).

### Tax Auditors

#### Sample

Overall, 30 Austrian tax auditors (13 of whom were female) who were on average 46.73 years (*SD* = 4.59) old participated in the study. Tax auditors reported their job experience, ranging from 6 years (3.3%) to 34 years (3.3%), with an average 20.70 years (*SD* = 7.52). Participants worked as tax auditors in three different eastern federal states of Austria; half were from the city of Vienna (53.3%) and half were from the country side (Styria: 26.7% and Lower Austria: 20.0%). Half of the tax auditors were responsible for auditing small and medium businesses, and the other half were responsible for auditing large businesses.

#### Procedure and Material

Access to 30 experienced tax officers was provided by the Austrian Ministry of Finance in 2013, which ensured that the participants were evenly distributed in terms of sex, urban vs. rural area, and area of responsibility. The interviews were conducted in the offices of the tax auditors by two interviewers. Interviews were semi-structured and lasted between 30 and 130 min. The interviews opened with a general question regarding tax auditors’ work, followed by questions about tax authorities’ potential to shape tax behavior (power of tax authorities), the role of taxpayers’ trust in the tax authorities, and the impact of power and trust on tax compliance. The interview questions were on taxes in general and did not specify a specific kind of tax. The interview materials can be found in the Supplementary Material, the transcripts can be found at osf.io/nv285/. No monetary or other form of remuneration was provided for participation, but the interviews were conducted during the tax auditors’ working hours.

### Analytical Procedure

The interviews with self-employed taxpayers and tax auditors were transcribed and analyzed using the qualitative analysis software NVivo (Qsr International Pty Ltd, 2010). The analysis followed an inductive as well as deductive approach. Data were analyzed using qualitative content analysis (QCA; Schreier, 2012), i.e., deriving codes from the data, as well as the extended slippery slope framework to provide codes. QCA is a method for systematically describing and conceptualizing the meaning of qualitative data, such as interviews, by categorizing parts of the material using a coding frame (Schreier, 2012). QCA is flexible in such as it is made to fit the material; thus, the coding categories are not purely theory-based but data driven. After data collection, two tax researchers read through the interviews, and build a coding frame based on the basic text and along the extended slippery slope framework. After the coding process, two other researchers who were also well-acquainted with tax research examined each of the categories, checking for homogeneity within and clear discrimination between them.

## Results

### Perception of Tax Authorities’ Power

In the following section, we present the results on perceived power. Table 1 summarizes the findings. The results indicated that tax auditors and taxpayers perceived the categories known in the literature, as well as mentioned some additional categories of authorities’ power.

**Table 1:** Results for tax authorities’ power.

	Categories	Examples
Coercive power		
Punishment power	Punishment/fines Audits Disclosure – negative Deadlines	*(Self-employed taxpayer #26, female, 40 years)* *“Somehow it is always a form of punishment [short laugh]”*
Reward power	Monetary: gain Non-monetary: praise Disclosure – positive Accommodation Active assistance	*(Tax auditor #21, male, 50 years)* *“Yes, this is actually a policy taken up more strongly in the last years by the financial administration, with which we want to reward the honest”*
Legtimate power		
Position power		*(Tax auditor #16, male, 49 years)* *“Well, I would see it in a way, that when I go out there, I want to leave the impression of a persisting instance, which ensures that the equality of taxation is adhered to”*
Expert power		*(Self-employed taxpayer #28, male, 60 years)* *“The tax authority is certainly the expert”*
Referent power	Image	*(Self-employed taxpayer #23, female, 43 years)* *“When I identify myself with the state and say ‘OK, the money is used for this or that and Austria is very worth living and therefore, we need taxes,’ this indeed increases tax honesty”*
Information power	Individual information Attain information oneself Publicity	*(Self-employed taxpayer #10, male 31 years)* *“Actually, that would be normal. That wouldn’t be so unusual, for example if you receive some kind of information per mail”*
Transparency		*(Self-employed taxpayer #13, male, 43 years)* *“The most important point would rather be transparency, more transparency on the part of the tax authority, so you know: the activity, the income, the expenditures, the taxes that you pay”*
Justice	Unfairness	*(Tax auditor #29, male, 50 years)* *“The person vis-à-vis cannot say, ‘I won’t declare parts of my income,’ he has to pay taxes, no matter what tax rate. It is simply about justice”*
Participation		*(Self-employed taxpayer #16, male, 34 years)* *“Of course there is the idea, that you can for example sort of decide, what taxes are used for, well. To a certain degree, entirely or third. I want to promote this, I want to promote this. Yes, this would simply be a marvelous way to see, where you want to get that to”*

*Direct translation*.

#### Coercive Power

##### Punishment power

As regards research questions 1a,b, the interviews illustrated that the tax stakeholders do perceive the categories of punishment power discussed in the literature (audits, fines, and public disclosure). Punishment power consisted of the categories of “punishment” (e.g., financial fine or imprisonment), “audits” (control mechanisms as well as monitoring), “negative disclosure” (public exposure of tax evaders), and a new category, “deadlines” (setting deadlines e.g., for taxpayers to submit documents). As one tax auditor claimed: “*And if he stood me up the fifth time, I write beneath, that I want a deadline.” Like, “Until that day you have to present the documents*” (Tax auditor #08, male, 41 years). It is of particular interest that “audits” played a significant role, as this category was mentioned more often than any other type of power measure. As one self-employed taxpayer (#13, male, 43 years) claimed: *“Well, without monitoring it is not working for sure.”*

#### Reward Power

Both tax auditors and taxpayers reported monetary and non-monetary rewards, as well as positive disclosure as aspects of tax authorities’ reward power (research question 2a). In particular, reward power consists of the monetary “gain” (e.g., tax reduction for taxpayers who pay on time – obviously, depending on legal constraints), non-monetary “praise” (e.g., “thank you letter” or positive feedback), “positive disclosure” (publicly praising honest taxpayers), and the new categories (research question 2b) of “accommodation” (the authority demonstrates goodwill, for instance when taxpayers can choose the date of an appointment), and “active assistance” (employers of the authorities go beyond their role, e.g., by providing helpful tips to honest taxpayers). Tax auditors reported that they reward taxpayers by praising them during audits: *“Yes, yes. So that is really important. People like that VERY much, when you praise them. I mean, everyone needs praise and even if they then [ask]: “So, is that alright like that?” “Yeah, you did an awesome job.”* (Tax auditor #12, female, 48 years). Some self-employed taxpayers noted that they perceived a lack of additional tax payment a reward in itself. Many self-employed taxpayers criticized the random application of rewards, believing that individual tax auditors offer tax reductions with varying levels of frequency. For instance, one self-employed taxpayers compared the auditing situation to a bazaar, whereby taxpayers and tax auditors are able to bargain about positive and negative reinforcement: “*And what makes me angry is that it became like a bazaar. So really like at a bazaar.”* (Self-employed taxpayer #23, female, 43 years).

Nevertheless, most of the self-employed taxpayers wanted the tax authority to have greater opportunities to reward desired behavior. As one self-employed (#06, male, age not indicated) said, *“Just introduce bonus systems. But this is already what we do today … what I have said before. Well, just bonus systems. Or MORE bonus systems.”*

#### Legitimate Power

Concerning research questions 3a,b, the interviews revealed that legitimate power consists of “position power” (the authority has the right to levy taxes), “expert power” (the authority and its employees are perceived to be experts), “referent power” (the authority has a positive image), “information power” (the authority is circulating information), “transparency” (all tax-related processes are transparent for the taxpayers), “justice” (the authority is treating all taxpayers fairly), and “participation” (taxpayers can take part in decision-making, e.g., how taxes are used). A tax auditor noted that transparency as information power is an important topic for self-employed taxpayers, especially when it comes to the action taken by the tax authority: *“You have to show a lot of transparency in what you are actually doing”* (Tax auditor #14, male, 38 years). Both types of self-employed taxpayers as well as tax auditors reported mechanisms of legitimate position power and information power more frequently than the other categories of legitimate power. A self-employed taxpayer (#01, female, 52 years) mentioned *“[…] that the tax authorities are legitimate, or simply that they are in the position, that they then use strategies, well they can implement this as strategies at taxpayers.”* One tax auditor emphasized that it was important to refer to the legal position of the institution of which he is a representative: “*There have to be rules and I have to tell the people “I am the tax authority’ when I come to them*” (Tax auditor #33, male, 46 years).

### Perception of Trust

Both self-employed taxpayers and tax auditors reported that taxpayers trust the tax authority implicitly as well as based on deliberation (Table 2). All categories of implicit and reason-based trust (Hofmann et al., 2014; Gangl et al., 2015) were mentioned in the interviews. In addition, new categories were found (e.g., implicit trust: personal support; reason-based trust: respect).

**Table 2:** Results for taxpayers’ trust in the tax authority.

	Category	Examples
Implicit trust	Blind-Automatic trust Sympathy and communication Empathy Experiences Shared values Equal partners Personal Support	*(Self-employed taxpayer #12, female, 27 years)* *“I think, that in Austria, a level of basic trust in the state system and therefore in the tax system exists”*
Reason-based trust	Common goal Internal factors Dependency External factors Respect	*(Self-employed taxpayer #07, female, 53 years)* *“No, no, I think (…) that doesn’t work with sympathy, that really only works due to facts and actions”*

*Direct translation*.

#### Implicit Trust

In line with research question 4a, both types of tax stakeholders reported that implicit trust consists of the categories *“*blind trust” (trusting the authority without thinking about it), “sympathy and communication” (taxpayers can communicate openly with employees of the tax authority), “empathy” (feeling of being understood by the authority), “shared values” (the authority and the taxpayers share the same values, or *weltanschauung*), and “equal stakeholders” (the tax authority and taxpayers interact at eye level). Furthermore, as regards research question 4b, “personal support” (taxpayers receive personal support from the employees of the tax authority) was mentioned as a new category. Some interviewees spoke quite generally of fundamental or basic trust (“Grundvertrauen”) in the tax system. As noted by a self-employed taxpayer: *“Well, I believe that in Austria there is a fundamental trust in the state system and therefore also in the system of taxation.”* (Self-employed taxpayer #12, female, 27 years). Personal support was considered important for implicit trust: “*So if you know the face behind the institution, that tells you: “Come here, we talk about it*” (Self-employed taxpayer” #11, female, 40 years), given that “*for the entrepreneur a personification of the tax office occurs*” (Tax auditor #30, male, 40 years). Many self-employed taxpayers refused to use the phrase “blind trust” when discussing their trust in the tax authority, given that it has a negative connotation, being associated with naivety toward the authorities’ actions. They preferred instead the phrase “automatic trust.” As one self-employed taxpayer (#03, female, 35 years) suggested: *“Well, simple blind, blind trust that shows a lot of naivety.”*

#### Reason-Based Trust

The interviews revealed that stakeholders do perceive the categories of reason-based trust discussed in the extended slippery slope framework (research question 5a). Reason-based trust consists of the categories “common goal” (tax authority and taxpayers share the same goals), “internal factors” (employees at the tax authority are competent, motivated, and benevolent), “dependency” (taxpayers depend on the tax authority and therefore trust the authority), and “external factors” (the perception of opportunities and dangers). Furthermore, as a novel category of reason-based trust (research question 5b), “respect” (respectful communication between the tax authority and the taxpayers) was mentioned. Tax auditors in particular claimed that interaction with taxpayers was most successful where there is mutual respect: *“As said before, the encounter. Every person needs to be respected”* (Tax auditor #21, male, 50 years), *“and taking them [the taxpayer] seriously and not talking deprecatory to them.”* (Tax auditor #09, male, 41 years). The interviews revealed that media reports concerning the unnecessary expenditure of taxes were perceived as important external factors that hinder the work of the tax authority. One tax auditor (#17, male, 49 years) stated: *“The media – you have said so already – […] that has an extreme effect.”* A second (Tax auditor #27, male, 54 years) argued: *“Based on different media reports this – how shall I say it – trust is nowadays, I believe, is not particularly high.”* Accordingly, self-employed taxpayers referred to negative media coverage of the topic of taxes. On the one hand, they referred to scandalous tax evasions by prominent people, but on the other hand they talked about the impression that their tax payments are wasted, e.g., “*Through the media you get to know how much is squandered*” *(self-employed taxpayer #11, female, 40 years).*

### Tax Compliance

Taxpayers as well as tax auditors reported a differentiated view of tax compliance. As shown in Table 3, both groups cited the relevance of behaviors such as “tax honesty,” “tax evasion,” and “tax avoidance” for compliance and non-compliance, which relate to categories that have already been discussed in the tax literature (research question 6a). However, “tax registration,” “timely filing,” “correct reporting,” “commercial,” and “aggressive tax planning” were not explicitly mentioned as distinct categories. Concerning research question 6b, “accepting tax behavior” and “stalling tax behavior” were identified as distinct categories. In the following section, we present the contents of all mentioned categories of tax behavior.

**Table 3:** Categories of tax compliance behavior mentioned in the interviews.

	Examples
Tax honesty	*(Self-employed taxpayer #05, male, 47 years)* *“The tax honesty in Austria is certainly higher; this is also an advantage”*
Tax evasion	*(Self-employed taxpayer #06, male, age not indicated)* *“If the authority takes advantage of it, then sooner or later it actually causes tax evasion, because somehow you always want to take revenge for giving you wrong advice or saddling you up with too many taxes”*
Tax avoidance	*(Tax auditor #15, female, 53 years)* *“And among the big corporations there are always the – not even loopholes – but legal possibilities to save taxes and this is actually, this is difficult”*
Accepting tax behavior	*(Tax auditor #10, female, 52 years)* *“Well (…) basically I follow the people – I honestly have to say that I rarely have difficulties – that the people basically are very cooperative and it works”*
Stalling tax behavior	*(Tax auditor #06, female, 49 years)* *“And on the other hand you find audits where the taxpayers block, where nothing is handed in”*

*Direct translation*.

#### Tax Honesty

Tax honesty was a relevant category of tax compliance for both self-employed taxpayers and tax auditors. Being tax honest was characterized as paying the full amount of the tax liability, in particular submitting all documents and transferring the correct amount of money to the tax authority on time. Self-employed taxpayers especially perceived tax honesty as significant in Austria: *“I guess that 90% of the people, without knowing the concrete numbers, are honest”* (Self-employed taxpayer #06, male, age not indicated).

#### Tax Evasion

When referring to tax evasion, all interviewees cited tax fraud. Of particular interest was the finding that the great majority thought of tax evasion as an intended behavior, with relatively few discussing tax evasion as “sloppy taxpaying” (“Schlampiges Steuerzahlen”). As a tax auditor (#26, female, 49 years) argued, *“There are firms that do this purely – how should I say – purely because of sloppiness.”* However, at least for some tax auditors the distinction between intended and observed tax evasion is important.

#### Tax Avoidance

Tax avoidance was considered in terms of being commercial rather than aggressive, and hence as a “normal,” legal and legitimate way of reducing the tax burden. Self-employed taxpayers generally talked very positively about this means of reducing the tax liability, often using the phrase “to save taxes” (“Steuern sparen”). For example, a self-employed taxpayer (#05, male, 47 years) claimed: “*The more someone possesses, the more he can employ someone to help save taxes*”; moreover, (#21, male, 53 years): *“BECAUSE in general the one who pays less tax is cleverer.”* A tax auditor (#08, male, 41 years) argued similarly: *“The citizen is like that, he tries to pay as less taxes as possible. That is in the nature of the human being.”*

#### Accepting Tax Behavior

Both self-employed taxpayers and tax auditors reported examples of “accepting tax behavior,” referring to taxpayers who accept tax authorities’ requests and behave cooperatively when interacting with the tax authority. Providing ordered and full materials, being accessible on the telephone, email or in person, and giving comprehensive answers when asked, were all considered examples of accepting tax behavior. A self-employed taxpayer (#08, female, 36 years) argued *“[…] that you be more precise, that you be perhaps also more punctual.”* A tax auditor mentioned (#07, male, 48 years) that *“If the counterpart is cooperative, it works pretty easily, it takes the simplest route.”*

#### Stalling Tax Behavior

In accordance with accepting tax behavior, stalling tax behavior has rarely been considered in previous tax literature. Tax auditors in particular spoke of taxpayers who fail to cooperate. Failing to provide all documents, intentionally creating complexity, or attempting to be inaccessible to the tax authorities comprised examples of stalling behavior. For instance: *“Why is this missing? Why is it not there? Or how long did someone have time? If you now say after a week he has said that he brings this and then it is not there. Or someone has postponed a meeting for the fourth time and then this occurs”* (Tax auditor #08, male, 41 years).

## Discussion

Research into tax compliance increasingly postulates that the quality of the relationship between tax authorities and taxpayers is an important factor that shapes tax compliance (Braithwaite, 2002; Feld and Frey, 2002; Kirchler, 2007). The results of the present study indicate that taxpayers and tax auditors indeed perceive power and trust categories as determinants of the relationship and use this categories to describe their tax relationship and tax compliance behavior. Thereby, the present study offers support for the assumptions of the extended slippery slope framework (Kirchler et al., 2008; Gangl et al., 2015) and allows a comprehensive and theoretical conceptualization of the determinants of the tax relationship and tax compliance behaviors. However, the present results also indicate that the tax stakeholders perceive categories that have scarcely been acknowledged in previous research. In addition to the well-known categories of power and trust, new sub-categories were identified that should be included in the extended slippery slope framework. The present research shows in particular, that the extended slippery slope framework as well as other theoretical models on the tax relationship (e.g., the responsive regulation theory) need to consider a larger variety of tax compliance behaviors. Thereby, the present research highlights research gaps and facilitates the generation of new research questions.

The results indicated that coercive power as a form of punishment pertains to audits, fines and negative disclosure (Allingham and Sandmo, 1972; Bø et al., 2014), as well as the new category of deadlines. Deadlines are not considered neutral, but rather as a means of enforcing compliance. To the best of our knowledge, empirical research on the effect of deadlines on the tax relationship, on tax compliance or on other forms of citizens’ compliance with the administration is rare. Thus, this represents an important starting point for future research and policy, because unilaterally established deadlines are a typical administrative instrument to influence citizens, and as the results here indicate, they may instigate the negative consequences of coercive power such as a reduction in trust and an increase in enforced motivation (Kirchler et al., 2008). Future quantitative research should examine the ways in which deadlines might be implemented to render them less aversive. For example, research on the effect of deadlines on student assignments has indicated that deadlines do not undermine intrinsic motivation if students are allowed to actively participate in the establishment of deadlines (Burgess et al., 2004), thus an increase in perceived legitimacy.

Reward power is related to monetary and non-monetary rewards and praise (Bazart and Pickhard, 2011; Simone et al., 2013; Koessler et al., 2016), as well as to the new categories “accommodation” (i.e., showing goodwill during the audit) and “active assistance” (i.e., helping honest taxpayers in accounting matters). It would appear that for many taxpayers, any administrative actions that are perceived as cooperative and as “not punishing” are considered a reward. In addition, our results demonstrate that taxpayers perceive rewards as something positive, and not as an additional form of coercion. However, again the combination with legitimate power might be important. The interviews indicated that rewards that result from arbitrary and non-transparent negotiations (e.g., like at a bazaar) can reduce trust in the administration and in turn preclude any intrinsic motivation to be honest (Deci, 1971; Frey, 1997). Future quantitative research should determine whether and under what conditions rewards can foster trust in the tax system.

The results for legitimate power indicated that (as expected) this is based on position, expert, referent and information power (French and Raven, 1959). As regards legitimate power, no new categories emerged. However, some related constructs of legitimate power were mentioned frequently, hence we summarized them into their own categories, including “justice,” “transparency” and “participation” (Feld and Tyran, 2002). It can be assumed that these are keywords, especially for taxpayers, which signal a legitimate tax system. Thus, the present research indicates that authorities possess numerous options to increase their perceived legitimacy.

In the tax relationship, implicit trust plays an important role, as tax stakeholders mentioned all known categories and spoke of a kind of “basic system trust” in the state and its institutions. In addition to empathy and perceived partnership, an interesting new category emerged: long-term “personal support.” The self-employed claimed to favor a person in the tax administration who can be considered personally responsible and an expert on their tax files. Although, personal assigned assistance (e.g., at unemployment agencies) is a standard procedure in other areas of public administration, this is not the case in tax administration. However exceptions include the specialized units of individual relationship managers for very wealthy taxpayers found in the United Kingdom (UK, National Audit Office, 2016). Future research should clarify whether a personal tax officer truly enhances trust and reduces stalling behavior, or whether in contrast this is considered an additional form of monitoring. In terms of personal support, numerous other (unintended) side effects must be considered, such as whether tax officers can remain neutral when they have known a taxpayer for a long time. Nonetheless, a personal support officer would undoubtedly represent a strong pillar for fostering a synergistic relationship between the tax authority and taxpayers. This “service” would change the culture in the administration, which is currently perceived by some as an anonymous, bureaucratic machine. Additionally, the current categories of implicit trust can be used in future quantitative studies utilizing recognition and speed tasks to examine whether implicit trust cues really lead to faster trust reactions than explicit trust cues.

Reason-based trust originates from a perception of a common goal, dependency, competence, motivation and benevolence; in sum, a supportive political environment (Castelfranchi and Falcone, 2010). The interviewees claimed that media reports play a crucial role in building (or compromising) reason-based trust, and so further quantitative research should be conducted to examine the positive and negative effects of media reports on tax compliance. “Respect,” as a new highlighted category of reason-based trust, is of course central to the tax relationship. However, to the best of our knowledge, the term has until now been used largely superficially (Feld and Frey, 2007) or considered a means of describing tax authorities’ respect for the legal rights of taxpayers (Murphy, 2004). In our interviews, respect meant mutual respect when interacting with each other, taxpayers’ respect for tax auditors and their expertise, and that tax auditors encounter taxpayers objectively and appreciatively. Future research should examine whether taxpayers’ respect has a real, positive influence on compliance, and whether tax auditors’ respect for taxpayers (e.g., for their hard work) can foster a synergistic relationship and build voluntary tax compliance.

The interviews also supported previous studies (Kirchler et al., 2003) that show that taxpayers and tax auditors alike talk about different categories of tax behavior. In addition to tax honesty, tax evasion and tax avoidance, the results indicate the importance of direct cooperative or non-cooperative contact between self-employed taxpayers and authorities as an aspect of tax compliance (“accepting behavior” and “stalling tax behavior”). Accepting tax behavior refers to all proactive supportive actions and service provisions on the side of the taxpayers that facilitate the quick and accurate determination of the real tax burden. In contrast, stalling tax behavior refers to all actions that jeopardize the work of the tax auditors and determination of the real tax burden. These new categories highlight the fact that some taxpayer behaviors are related to more costly administrative burdens than others. It should be noted that these categories are distinct from administrative (Organisation for Economic Co-operation and Development [OECD], 2001) or reporting compliance (Brown and Mazur, 2003), because they concern aspects such as being available or not intentionally creating complexity in bookkeeping, which is different from filing or submitting material on time. However, in spite of their obvious relevance, to the best of our knowledge, little research exists regarding the strategies that influence taxpayers’ accepting or stalling behavior in collaboration with tax authorities. We believe that this finding offers important directions for future research. Another interesting finding was that tax auditors spoke of unintentional sloppiness as a factor behind tax evasion. Future research could examine whether lack of knowledge or motivation leads to sloppiness in tax filing and paying and how tax auditors can distinguish intention from sloppiness. Whereas intention needs compulsory action, sloppiness (for example, also because of the complex tax system) can be tolerated by tax auditors and needs supportive service related actions. Empirical examination of the fostering of ordered and accepting tax behavior and reducing stalling tax behaviors has considerable potential in reducing the administrative costs of collecting taxes.

Although the current approach has its merits, there are some limitations that must be considered in future research. The current research takes into account only one country with a relatively high level of trust in public institutions (Alm and Torgler, 2006; Schneider et al., 2010). The present results are, thus, most generalizable only to other European countries with similar tax morale, especially Germany, which shares a similar legal and cultural background with Austria (see Supplementary Material for details on the Austrian tax system). Thus, the present results might not fully capture the reality of developing countries (and others) that have large populations of non-filers (Gangl et al., 2017). Also the view of additional stakeholders, in particular the tax advisors is missing in the present research. In addition, our results are unlikely generalizable to large international corporations. Based on our sample selection, our results apply to self-employed taxpayers and less likely to employed taxpayers. However, self-employed taxpayers who have to submit their taxes personally likely have more experiences with the tax authority and with taxpaying compared to employed taxpayers who’s taxes are deducted automatically. Due to our aim to investigate a large diversity of views and due to the qualitative research design with a relative small sample, it is not possible to determine whether the perceptions of tax auditors and taxpayers differ. We find that both stakeholder groups hold similar perceptions. They gave similar examples of power, trust and tax compliance. Although, we do not find indication of clear differences, we suggest that future quantitative research (e.g., example questions can be found in the Supplementary Material) targets different views of stakeholders and gives priority to similarities and differences to understand possible misunderstands and conflicts. We used the extended slippery slope framework to review the literature and to categorize the interview data. Thus, applying different theoretical models might lead to other categories. However, given that the extended slippery slope framework builds on established theories of taxation (Allingham and Sandmo, 1972; Braithwaite, 2002; Kirchler, 2007), power (French and Raven, 1959; Tyler, 2006), trust (Castelfranchi and Falcone, 2010), regulatory relationships (Ouchi, 1979; Adler, 2001), and cognitive processing (Kahneman, 2003), we are confident that our results are valid. Finally, as most qualitative studies also our study is based on a relative small, non-randomly selected sample and does not allow generalizable conclusions and hypotheses testing. Nonetheless, the present qualitative study is the first which rigorously investigates both, the tax auditors and taxpayers. Therefore, we are confident that the present results are a fruitful starting point for future quantitative research on tax compliance with larger samples from different countries.

The current qualitative interview study can fuel further quantitative research. The categories found in these interviews can be used to develop more accurate measurement instruments (see Supplementary Material for example questions and scales) to evaluate tax administrative policies and to analyze the relationship between different determinants of the tax relationship and tax behaviors. Further, an important influencing factor of the perception of power and trust, as well as tax behavior might be the frequency of taxpayers’ contacts with the tax authority. Also the employment of tax advisors, as intermediaries between self-employed taxpayers, and the tax authority, may have a significant impact on the taxpayers’ perception of the tax authority. Future studies should test the causal link between relationship determinants and tax compliance behaviors. Existing studies on coercive and legitimate power have not considered deadlines, different forms of reward power or personal support, all of which may be considered determinants of compliance. Some of the “known” categories such as public disclosure continue to require further empirical investigation. Empirical evidence regarding the effects (and side effects) of negative disclosure through “black lists” and public shaming is also limited; maybe shaming only effects middle class but not wealthy tax evaders (Lenter et al., 2003; Perez-Truglia and Troiano, 2015; Casal and Mittone, 2016). Empirical evidence regarding the effect of positive disclosure (e.g., the Fairtax mark) is especially rare.

The main advantage of the current paper is that the perspectives of both individual citizens and authorities were considered. Based on this strong empirical grounding, the present outcomes have considerable practical relevance. They indicate that the relationship between tax authorities and taxpayers is of inherent importance for compliance. Taxpayers do not simply respond and tax auditors do not simply use command and control, but rather demonstrate a sophisticated understanding and nuanced behaviors when interacting with one another. However, in most countries the training of tax auditors and the approach of tax authorities continue to focus on “hard” auditing and monitoring skills, whereas the soft skills used to shape relationships with taxpayers are neglected. The present results present a summary of instruments of coercive power, legitimate power, reason-based trust, and implicit trust and can be used to develop strategies to improve the relationship between authorities and taxpayers, and training programs for tax auditors aiming to improve their communication skills when interacting with taxpayers. For instance, in workshops, setting deadlines could be trained such that their perceived coerciveness is reduced.

A significant trend in tax administration is digitalization and automating, as well as reducing the personal interaction between tax authorities and taxpayers (Kochanova et al., 2017). Based on the results presented here, in particular on the appreciation of personal support and respect as novel categories of implicit and reason-based trust, we argue that the resources invested in cooperative relationship programs should be increased. Without doubt, digital services that enhance tax handling for taxpayers are required. However, relying solely on a machine-mediated interaction between the tax authorities and taxpayers, with the aim to reduce personalized service costs, bears the risk that tax behavior degenerates to a merely rational calculating task. The social dimension of paying one’s contribution must not be neglected.

## Ethics Statement

This study was carried out in accordance with the recommendations of Declaration of Helsinki (7th revision, 2013) and local ethical guidelines for studies with human participants (including approval by an institutional review board) at the Faculty of Psychology of the University of Vienna with written informed consent from all subjects. All subjects gave written informed consent in accordance with the Declaration of Helsinki. The protocol was approved by the institutional review board at the Faculty of Psychology.

## Author Contributions

EH, KG, and EK planned the study. KG and BH collected and analyzed the data. KG, BH, EH, and EK wrote the manuscript.

## Conflict of Interest Statement

The authors declare that the research was conducted in the absence of any commercial or financial relationships that could be construed as a potential conflict of interest.

## Footnotes

1 fairtaxmark.com
