# Supplementary material for: The Relationship Between Austrian Tax Auditors and Self-Employed Taxpayers: Evidence From a Qualitative Study
Source: Front Psychol. 2019 May 13;10:1034. doi: 10.3389/fpsyg.2019.01034 (PMC6526770; doi:10.3389/fpsyg.2019.01034)
Supplement: Supplementary file 1 [file Data_Sheet_1.docx]

**Supplementary material**

The Relationship between Austrian Tax Auditors and Self-Employed Taxpayers: Evidence from a Qualitative Study

Background information on the Austrian tax system...………………………….....page 2

Interview guideline and short-survey for self-employed taxpayers………………..page 4

Interview guideline and short-survey for tax auditors……………………………..page 19

Transcripts of all interviews……………………………………………………….page 29

Example of a measurement instrument based on the current findings…………….page 29

**Background information on the Austrian tax system**

Austria is a central European and German-speaking country with approximately 9 million inhabitants. In 2016 about 473.000 Austrians were self-employed which equals to a self-employed quote of 11.2 percent (Germany has a quote of 10.0 percent). Self-employed are approximately five times wealthier than employed Austrians (Humer, Moser, Ertl, & Kilic, 2014); 42 percent of Austrian millionaires are self-employed. Thus, self-employed, also because of their increased possibilities to evade taxes, are an important population concerning the total tax return.

The European Commission estimates that the VAT-gap in 2015 (evaded VAT tax) for Austria is 8.24% (for Germany: 9.56%; Figure 1). The size of the shadow economy is estimated to be 9.16% (for Germany: 14.70, Schneider & Bühn, 2017). To detect tax evasion, third-party information is an important tool of the Austrian tax authorities, however the Austrian Ministry of Finances does not share details on its use or prevalence. The audit probability in Austria is determined by many factors such as size of the company, legal status, past tax behavior, branch of a company, external information and information from the PACC (Predictive Analytics Competence Center) which analyses companies with a specialized software to select them for audits. During an audit the Austrian tax authorities select from a series of instruments such as investigations, VAT-audit, or company tax audit. Concerning its legal and administrative tax system, Austria is most similar to Austria.


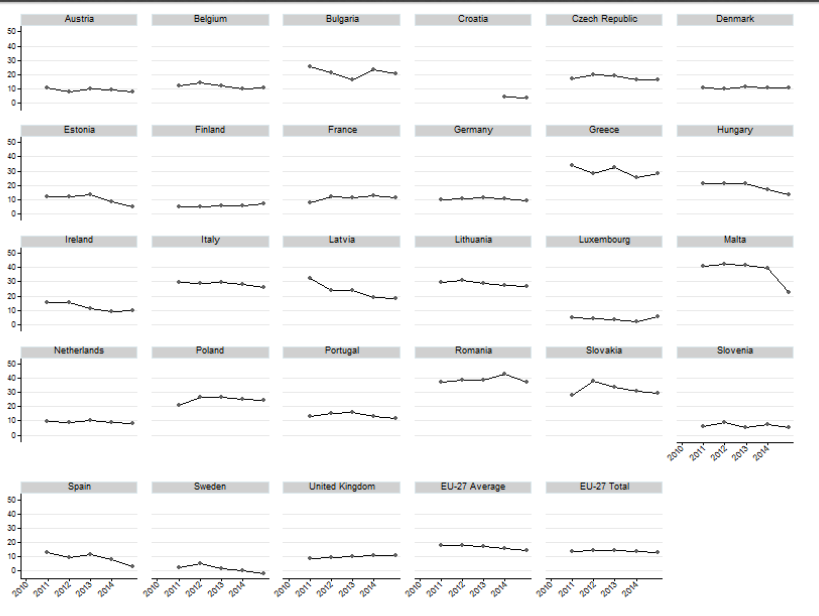


Figure 1: European Commission Estimations of the VAT-gap (page 18)

Resources on Austria:

1. [**http://www.statistik.at/web_de/statistiken/menschen_und_gesellschaft/arbeitsmarkt/erwerbstaetige/selbstaendige_mithelfende/index.html**](http://www.statistik.at/web_de/statistiken/menschen_und_gesellschaft/arbeitsmarkt/erwerbstaetige/selbstaendige_mithelfende/index.html)
2. [**http://wko.at/statistik/eu/europa-selbstaendigenquote.pdf**](http://wko.at/statistik/eu/europa-selbstaendigenquote.pdf)
3. [**https://ec.europa.eu/taxation_customs/business/tax-cooperation-control/vat-gap_en**](https://ec.europa.eu/taxation_customs/business/tax-cooperation-control/vat-gap_en)
4. Humer, S., Moser, M., Schnetzer, M., Ertl, M., & Kilic, A. (2014). Sozioökonomische Charakteristika der Vermögensverteilung in Österreich. Report of the Economic Champer in Austria (WKO).
5. Schneider, F. & Buehn, A. (2017). Shadow economy: Estimation methods, problems, results and open questions. Open Economics, 1, 1-29.

**Interview guideline for self-employed taxpayers**

| German Original | English Translation |
| --- | --- |
| **Informationen zum Interview**  Wir führen im Rahmen eines Forschungsprojektes an der Universität Wien Interviews durch und untersuchen die Meinungen von Selbstständigen zu Steuern.  Unser Gespräch wird mit einem Diktiergerät aufgezeichnet, aber Ihre Daten werden selbstverständlich anonym und vertraulich behandelt. Das gesamte Gespräch wird gleich nach der Transkription wieder gelöscht. Hierfür würde ich Sie bitten, diese Erklärung zu unterschreiben.  Wir freuen uns an Ihren Erfahrungen teilhaben zu können und bedanken uns herzlich, dass Sie sich Zeit für uns genommen haben.  Dauer: ungefähr 1 ½ h  Uns ist es wichtig, dass das Interviews nicht unterbrochen wird, aus diesem Grund würde ich Sie bitten das Handy ausschalten.  Wir starten zunächst mit ein paar kurzen Fragen zu Ihrer Person und Ihrer Tätigkeit und stellen im weiteren Verlauf Fragen zu Ihrer Meinung zu Steuern. Wichtig ist, dass es keine richtigen und falschen Antworten gibt. Antworten Sie einfach „aus dem Bauch“ heraus.  Legende (für den Interviewer):  (I) implizites Vertrauen  (R) rationales Vertrauen  (C) coercive Macht  (L) legitime Macht  Schwarze und kursive Schrift: was wir zusätzlich erwähnen können | **Information on the Interview**  In the context of a research project at the University of Vienna, we are conducting interviews in order to investigate the opinions of self-employed on taxes.  Our conversation is being recorded, but of course your data will be treated anonymously and confidentially. The whole conversation will be deleted right after it has been transcribed.  Therefore, please sign this declaration.  We are happy you are sharing your experiences with us and would like to thank you cordially for taking your time for us.  Duration: ca. 1,5 h  It is important for us, that the interview will not be interrupted. Therefore, we kindly ask you to turn off your phone.  We are going to start with a few questions regarding your person and your job and will proceed to ask questions concerning your opinion on taxes. Importantly, there are no right or wrong answers. Just answer intuitively.  Legend (for the interviewer):  (I) implicit trust  (R) rational trust  (C) coercive power  (L) legitimate power  Black and italic: could be mentioned additionally |

| German Original | English Translation |
| --- | --- |
| **Kurzfragebogen**  **Geschlecht:**   - Männlich - Weiblich   **Wie lange sind Sie bereits selbstständig tätig (in Jahren)? ________**  **Wie alt sind Sie?** *(mit Feingefühl fragen, eventuell erst am Ende der demografischen)* **______**  **Haben Sie eigene Angestellte/ MitarbeiterInnen?**   - Ja - Nein   **Wenn ja, wie viele? _______** | **Short Questionaire**  **Gender:**   - male - female   **How long have you been self-employed (duration in years)?______**  **How old are you?** *(when asking be sensitive, maybe ask at the end of demographics)* **______**  **Do you have employees?**   - Yes - No   **If yes, how many? _____** |

| German Original | English Translation |
| --- | --- |
| 1. Einstiegsfragen  - In welcher Weise beschäftigen Sie sich als Selbstständige(r) mit dem Thema Steuern? - Beschäftigen Sie einen Steuerberater für Ihre Steuererklärung?   - Ja   - Nein - Die Steuerbehörde ist ja auch Teil dieses Themas Steuern. Was ist Ihre Meinung von der Steuerbehörde? | 1. Introductory Questions  - As a self-employed, how do you deal with the topic taxes? - Do you hire a tax consultant for your tax return?   - Yes   - No - The tax authority is part of the subject taxes. What is your opinion on the tax authority? |

| German Original | English Translation |
| --- | --- |
| 1. Macht auf Verhalten   Uns interessieren auch die Maßnahmen von Steuerbehörden:   - Welche Strategien setzt die Steuerbehörde ein, um die Steuereinnahmen zu erhöhen? *(wenn nichts kommt, nachfragen:)* Welche persönlichen Erfahrungen haben Sie gemacht? *(wenn nichts kommt, nachfragen:)* Welche Möglichkeiten hat sie noch? Welche Möglichkeiten kennen Sie vielleicht von anderen Steuerzahlern oder von anderen Ländern?   *(Wenn eine Art der Macht angesprochen wird, nachfragen):* Wie sieht das konkret aus?  Ich möchte Ihnen gerne Einflussstrategien aus einem anderen Bereich vorstellen. Überlegen Sie bitte, ob diese Strategien auch im Steuerbereich angewendet werden könnten. Wenn Sie z.B. an die Einflussmöglichkeiten eines Unternehmens denken, so gibt es verschiedene Möglichkeiten wie das Unternehmen Einfluss auf seine Mitarbeiter nehmen kann. Ich würde Sie bitten, sich jeweils den folgenden Text durchzulesen.   - (C/coercive) Ein Unternehmen und die Unternehmensleitung kann mit Hilfe von Bestrafungen Einfluss auf seine Mitarbeiter nehmen, indem es Druck ausübt, seine Mitarbeiter kontrolliert und mit Sanktionen droht, wie beispielsweise einer Versetzung, Kündigung oder Nichtauszahlung eines Bonus.   Kann es diese Möglichkeit der Einflussnahme auch im Steuerkontext geben? *(Wenn nichts kommt, Kontrolle, Androhen von Strafen ansprechen)*  Wie könnte das aussehen? (bzw.: Warum nicht?)   - (C/reward) Ein Unternehmen und die Unternehmensleitung haben die Möglichkeit, mit Hilfe von Belohnungen Einfluss auf seine Mitarbeiter zu nehmen. So sind etwa eine Gehaltserhöhung, aber auch Lob und wertschätzende Worte Einflussmöglichkeiten, ebenso wie das Abwenden des Verlusts des Arbeitsplatzes oder die Eröffnung von Aufstiegschancen.   Kann es diese Möglichkeit der Einflussnahme auch im Steuerkontext geben? *(Wenn nichts kommt, Vergünstigungen, Entgegenkommen seitens Steuerprüfer ansprechen)*  Wie könnte das aussehen? (bzw.: Warum nicht?)   - (L/legitimate) Das Unternehmen und seine Leitung hat Einfluss auf die Mitarbeiter aufgrund der rechtmäßigen Position des Unternehmens gegenüber den Mitarbeitern, die die Stellung des Unternehmens nicht in Frage stellen.   Kann es diese Möglichkeit der Einflussnahme auch im Steuerkontext geben? *(Wenn nichts kommt, Überzeugung vom Steuersystem und Legitimation ansprechen)*  Wie könnte das aussehen?  (bzw.: Warum nicht?)   - (L/reference) Ein Unternehmen und die Unternehmensleitung hat die Möglichkeit Einfluss auf seine Mitarbeiter zu nehmen, wenn diese sich mit dem Unternehmen identifizieren und ihm gegenüber das Gefühl von Verbundenheit und Wertschätzung empfinden.   Kann es diese Möglichkeit der Einflussnahme auch im Steuerkontext geben? *(Wenn nichts kommt, Identifizieren mit dem Steuersystem ansprechen)*  Wie könnte das aussehen? (bzw.: Warum nicht?)   - (L/expert) Ein Unternehmen und die Unternehmensleitung hat Einfluss auf seine Mitarbeiter, da die Unternehmensleitung Experten auf ihrem Gebiet sind.   Kann es diese Möglichkeit der Einflussnahme auch im Steuerkontext geben? *(Wenn nichts kommt, Instruieren von Steuerprüfer ansprechen)*  Wie könnte das aussehen? (bzw.: Warum nicht?)   - (L/information) Eine Einflussmöglichkeit des Unternehmens kann darin bestehen, dass das Unternehmen Informationen weitergibt, welche die Mitarbeiter dazu veranlassen, ihre Aufgaben im Sinne des Unternehmens zu erledigen.   Kann es diese Möglichkeit der Einflussnahme auch im Steuerkontext geben? *(Wenn nichts kommt, Finanzonline nennen)*  Wie könnte das aussehen? (bzw.: Warum nicht?)  **C+L – Steuerehrlichkeit**   - Wir haben jetzt über (*z.B.: Androhen von Konsequenzen, Druck*) gesprochen. Wenn Sie als selbstständige(r) SteuerzahlerIn seitens der Steuerbehörde damit konfrontiert sind, wie würde das ihr Steuerverhalten beeinflussen? (bzw.: Warum nicht?) - Wir haben auch über (z.B.: *Überzeugungskraft, Fachwissen*) gesprochen. Wenn Sie als selbstständiger Steuerzahler seitens der Steuerbehörde damit konfrontiert sind, wie würde das ihr Steuerverhalten beeinflussen? (bzw.: Warum nicht?)   ZU DEN ZWEI: Gibt es zu diesen Punkten noch Fragen von eurer Seite? | 1. Power on Behaviour   We are interested in the actions of tax authorities:   - Which strategies does the tax authority use in order to increase tax revenue? *(if no answer, ask:)* Which personal experiences have you had? *(if no answer, ask:)* Which possibilities does it have? Which other possibilities do you maybe know from other taxpayers or other countries?   *(If some form of power is adressed, ask:)* How does it look exactly?  I would like to introduce you to influencing strategies from another domain. Please think about if those strategies could be applied in the tax domain. If you think of a company´s possibilities to influence, there are several possibilities on how the company can influence its employees. Please read the following texts.   - - (C/coercive) A company and the management can influence their employees with the help of punishment by exerting pressure, controlling the employees and threaten to impose sanctions, for example a relocation, termination or not paying bonus.   Can this possibility to influence also be used in the context of taxes? *(If no answer, mention control/threat of punishment)*  How could this look like?  (respectively: Why not?)   - - (C/reward) A company and the management have the possibility to influence their employees with the help of reward. A salary increase, but also praise and appreciative words are possibilities to influence, also averting the loss of the job or disclosing to opportunity for promotion.   Can this possibility to influence also be used in the context of taxes? *(If no answer, mention perks, concession of the tax auditor)*  How could this look like?  (respectively: Why not?)   - - (L/legitimate) The company and its management can influence their employees due to its legal position towards employees, who do not question the company´s position.   Can this possibility to influence also be used in the context of taxes? *(If no answer, mention conviction of the tax system and legitimation)*  How could this look like?  (respectively: Why not?)   - - (L/reference) A company and its management have the possibility to influence their employees when they identify with the company and have the feeling of affinity and appreciation towards it.   Can this possibility to influence also be used in the context of taxes? *(If no answer, mention identification with the tax system)*  How could this look like?  (respectively: Why not?)   - - (L/expert) A company and its management have the possibility to influence their employees, because the management are experts on their field.   Can this possibility to influence also be used in the context of taxes? *(If no answer, mention instruction of the tax auditor)*  How could this look like?  (respectively: Why not?)   - - (L/information) One possibility of the company to influence can be, that the company shares information, which causes the employees to handle their tasks in the interest of the company.   Can this possibility to influence also be used in the context of taxes? *(If no answer, mention finance-online tool)*  How could this look like?  (respectively: Why not?)  **C+L – Honesty in taxation**   - - We have now talked about *(e.g.: threat of punishment, pressure).* If you as a self-employed be confronted with that by the tax authority, how would it influence your behaviour on taxes (respectively: Why not?)   - We also talked about *(e.g.: persuasiveness, expert knowledge)*. If you as a self-employed be confronted with that by the tax authority, how would it influence your behaviour on taxes (respectively: Why not?)   REGARDING THOSE TWO: Are there any more questions on these topics from your side? |

| German Original | English Translation |
| --- | --- |
| 1. VERTRAUEN (auf Verhalten)   Bis jetzt haben wir über verschiedene Maßnahmen gesprochen, die dazu führen können, dass Personen mit Institutionen kooperieren. Darüber hinaus gibt es aber natürlich noch andere Gründe für Kooperation, wie beispielsweise Vertrauen in diese Institutionen.   - Vertrauen Sie der Steuerbehörde?   - Warum (nicht)? (*Wenn „vertraue nicht“ kommt, genauer fragen, ob Sie ihnen Misstrauen entgegenbringen oder nur nicht vertrauen“ -> das gilt auch für die Fragen unten)*   Können andere Steuerzahler andere Gründe haben um zu vertrauen?  Wie muss die Steuerbehörde sein, um ihr vertrauen zu können?   - (R) Wenn Sie nun wieder an den Unternehmenskontext denken: Ein Grund für das Vertrauen von Mitarbeiter in ihr Unternehmen könnte sein, dass sie das Unternehmen als kompetent, motiviert und ihnen gegenüber als wohlwollend wahrnehmen. Kann es das auch zwischen Selbstständigen und der Steuerbehörde geben? - Warum (nicht)? Wie könnte das aussehen?   *(Nachfragen, falls nicht auf jeden der drei Aspekte eingegangen wird: Sie sprechen jetzt nur von X, wie verhält es sich mit (kompetent/motiviert/wohlwollend)?*   - (R) Wenn Sie nun wieder an den Unternehmenskontext denken: Mitarbeiter können dem Unternehmen auch vertrauen, weil dieses dasselbe Ziel verfolgt wie sie.   Kann es das auch zwischen  Selbstständigen und der Steuerbehörde  geben?   - Warum (nicht)? Wie könnte das aussehen? - (R) Wenn wir wieder zurück zum Unternehmensbeispiel gehen: Auch wenn das Unternehmen und die Unternehmensleitung wohlwollend und kompetent sind, kann es immer äußere Einflüsse geben, welche die Erreichung des gemeinsamen Ziels stören. Ein Unternehmen und seine Mitarbeiter können das gemeinsame Ziel beispielsweise aufgrund von Wirtschaftskrisen oder eine Änderung der Gesetzeslage nicht erreichen. Kann es das auch zwischen Selbstständigen und der Steuerbehörde geben? *(zum Beispiel nicht ausreichend Geld vorhanden, die Politik setzt sich nicht ein)* - Warum (nicht)? Wie könnte das aussehen? - (R) Um wieder zurück zu unserem Unternehmenskontext zu kommen: Mitarbeiter kooperieren möglicherweise auch deshalb mit dem Unternehmen, weil Sie vom Unternehmen in gewisser Weise abhängig sind. Kann es das auch zwischen Selbstständigen und der Steuerbehörde geben? - Warum (nicht)? Wie würde sich das äußern?   (I) Es gibt auch Vertrauen in andere ohne darüber nachzudenken, Mitarbeiter können einem Unternehmen beispielsweise auch blind vertrauen, sozusagen: automatisch vertrauen.   - Kann es das auch zwischen Selbstständigen und der Steuerbehörde geben? - Warum (nicht)? Wie könnte das aussehen? - (I) Automatisches Vertrauen von Mitarbeitern in das Unternehmen entsteht aufgrund von positiven Erfahrungen mit dem Unternehmen. Kann es das auch zwischen Selbstständigen und der Steuerbehörde geben?   - Was wären das für positive Erfahrungen? - (I) Wenn Sie nun wieder an den Unternehmenskontext denken: Für das Vertrauen zwischen Mitarbeitern und dem Unternehmen kann auch Sympathie eine Rolle spielen. Kann es das auch zwischen Selbstständigen und der Steuerbehörde geben? - Warum (nicht)? Wie könnte das aussehen? - (I) Mitarbeiter könnten dem Unternehmen auch vertrauen, weil das Unternehmen dieselben Werte vertritt und dieselbe Weltanschauung teilt wie die Mitarbeiter. Kann es das auch zwischen Selbstständigen und der Steuerbehörde geben? - Warum (nicht)? Wie könnte das aussehen? - (I) Wenn wir wieder zurück zum Unternehmensbeispiel gehen: Mitarbeiter könnten dem Unternehmen auch vertrauen, weil sie das Gefühl haben, offen mit der Unternehmensleitung reden zu können. Kann es das auch zwischen Selbstständigen und der Steuerbehörde geben? - Warum (nicht)? Wie könnte das aussehen? - Wir haben jetzt darüber gesprochen, dass Vertrauen in die Steuerbehörde entweder automatisch entstehen kann, oder weil dieselben Ziele verfolgt werden. Wenn Sie als selbstständiger Steuerzahler der Steuerbehörde vertrauen, wie würde das ihr Steuerverhalten beeinflussen? (bzw.: Warum nicht?)   ZU DEN ZWEI: Gibt es zu diesen Punkten noch Fragen von eurer Seite? | 1. Trust (in behaviour)   So far we have talked about different measures, which may lead to people cooperating with institutions. Beyond that, there are other reasons for cooperation, for example trust in these institutions.   - Do you trust the tax authority?   - Why (not)? *(If answer is “do not trust”, ask more precisely, whether you have suspicion against it, or only mistrust them 🡪 applies for every question below )*   May other taxpayers have different reasons to trust?  How does the tax authority have to be, in order to be trusted?   - (R) If you may think again in the context of companies: A reason for the trust of your employees in the company could be, that they perceive the company as competent, motivated and benevolent towards them. Might this exist between self-employed and the tax authority?   - Why (not)? How could this look like?   *(Ask again, if it has been answered only to one the three aspects: You speak only about X, how about (competent/motivated/benevolent)?*   - (R) If you may think again in the context of companies: employees may also trust the company, because it pursues the same goal as them.  Might this exist between self-employed and the tax authority?   - Why (not)? How could this look like? - (R) Going back to the example of a company: Even if the company and the management is benevolent and competent, there might be external influences, which disturbs the achievement of the common goal. A company and its employees might not achieve their goal due to for example an economic crises or a change in legislation. Might this exist between self-employed and the tax authority? *(e.g. there is not enough money, politics does not support)*   - Why (not)? How could this look like? - (R) Coming back to the context of companies: employees might cooperate with the company, because they are in a way dependent on the company. Might this exist between self-employed and the tax authority?   - Why (not)? How would this   manifest itself?  (I) There is also trust in others without thinking about it, for example employees might trust a company blindly, trust automatically so to speak.   - Might this exist between self-employed and the tax authority?   - Why (not)? How could this look   like?   - (I) Automatic trust of employees in the company results from positive experiences with the company. Might this exist between self-employed and the tax authority?   - What kind of positive experiences would that be? - (I) If you think back now to the context of companies: concerning the trust between employees and the company, sympathy might play a role. Might this exist between self-employed and the tax authority?   - Why (not)? How could this look like? - (I) Employees might also trust companies, because the company shares the same values and ideology like the employees. Might this exist between self-employed and the tax authority?   - Why (not)? How could this look like? - (I) Coming back to the example of the company: employees might also trust the company, because they have the feeling, they can openly talk the company´s management. Might this exist between self-employed and the tax authority?   - Why (not)? How could this look like? - We have now talked about, that trust in the tax authority might emerge automatically, or because the same goals are pursued. If you as self-employed taxpayer trust the tax authority, how would this influence your behaviour on taxes? (respectively: Why not?)   REGARDING THOSE TWO: Are there any more questions on these topics from your side? |

| German Original | English Translation |
| --- | --- |
| B. STEUERKLIMATA - WECHSELSPIEL VON MACHT UND VERTRAUEN  Ich würde Ihnen nun gerne verschiedene Situationen schildern, die die Beziehung zwischen Steuerbehörde und Steuerzahler beschreiben können und Ihnen anschließend Fragen dazu stellen. Ich würde Sie nun bitten, sich folgende Situation vorzustellen: *(Anfang der Szenarien anpassen, je nachdem mit welchem begonnen wird)*  AUSBALANCIEREN:   \| - A S C \| - S A C \| - C A S \| \| --- \| --- \| --- \| \| - A C S \| - S C A \| - C S A \|   Antagonistisches Klima (CP: ↑, LP: ↓, RBT: ↓, IT:0)  Sie wohnen in einem Land, indem sich Steuerbehörde und Steuerzahler gegenseitig argwöhnisch beobachten. Die Steuerzahler werden von der Steuerbehörde als Verbrecher gesehen, die sich jederzeit nur bereichern wollen und jede Möglichkeit nutzen, Steuern zu hinterziehen. Die Steuerzahler fühlen sich hingegen verfolgt und nehmen die Steuerbehörde als Jäger wahr. Die Beziehung zwischen Steuerbehörde und Steuerzahler ist gekennzeichnet durch unbarmherziges und rücksichtsloses Verhalten.   - Wenn Sie jetzt noch einmal an die Einflussmöglichkeiten denken, von denen wir vorher gesprochen haben, wobei Einfluss einerseits mittels Bestrafungen ausgeübt werden kann, andererseits aber auch durch Fachwissen und dem Geben von Informationen. Wenn Sie jetzt an die geschilderte Situation denken, übt die Steuerbehörde in dieser Situation Einfluss aus? Wenn ja, wie? (bzw. Warum nicht) *(Nur nachfragen, wenn nicht hypothesenkonform geantwortet wird)* - Wenn Sie jetzt noch einmal an die vorherigen Fragen zu Vertrauen in Institutionen denken: Jemand kann dem Gegenüber einerseits automatisch vertrauen, ohne darüber nachzudenken, andererseits kann jemand dem Gegenüber auch vertrauen, weil er weiß, dass dieser kompetent ist und dieselben Ziele verfolgt. Vertrauen die Steuerzahler in dieser Situation der Steuerbehörde? Wenn ja, wie zeichnet sich das Vertrauen aus? (bzw. Warum nicht?)   Change: Stellen Sie sich nun vor, dass fünf Jahre vergangen sind und Sie immer noch in diesem Land leben. Es ist in dieser Zeit zu Veränderungen gekommen und die Situation zwischen Steuerzahlerinnen und Steuerbehörde hat sich geändert:  Serviceklima (CP: ↓, LP: ↑, RBT: ↑, IT: ↓)  Sie wohnen in einem Land, indem Steuerbehörde und Steuerzahler auf Basis von Regeln und Standards zusammenarbeiten und sich wie ein Unternehmen und seine Kunden verhalten. Die Beziehung zwischen Steuerbehörde und Steuerzahler ist vom Servicegedanken geprägt.   - *(Abkürzen, wenn S nicht als erstes Szenario)* Wenn Sie jetzt noch einmal an die Einflussmöglichkeiten denken, von denen wir vorher gesprochen haben, wobei Einfluss einerseits mittels Bestrafungen ausgeübt werden kann, andererseits aber auch durch Fachwissen und dem Geben von Informationen. Wenn Sie jetzt an die geschilderte Situation denken, übt die Steuerbehörde in dieser Situation Einfluss aus? Wenn ja, wie? (bzw. Warum nicht) *(Nur nachfragen, wenn nicht hypothesenkonform geantwortet wird)* - Wenn Sie jetzt noch einmal an die vorherigen Fragen zu Vertrauen denken: Jemand kann dem Gegenüber einerseits automatisch vertrauen, ohne darüber nachzudenken, weil der andere beispielsweise sympathisch ist, andererseits kann jemand dem Gegenüber auch vertrauen, weil er weiß, dass dieser kompetent ist und dieselben Ziele verfolgt. Wenn Sie jetzt an die geschilderte Situation denken, vertrauen die Steuerzahler in die Steuerbehörde? Wenn ja, wie zeichnet sich das Vertrauen aus? (bzw. Warum nicht?) - Welche Ereignisse könnten vorgefallen sein, die zu dieser Veränderung in der Beziehung zwischen Steuerzahler und Steuerbehörde geführt haben?   Change: Stellen Sie sich vor, dass weitere fünf Jahre vergangen sind und Sie immer noch in diesem Land leben. Es ist in dieser Zeit wieder zu einigen Veränderungen gekommen und auch die Situation zwischen Steuerzahlerinnen und Steuerbehörde hat sich wieder geändert:  Confidence Klima (CP: 0, LP: ↓, RBT: ↓, IT: ↑)  Stellen Sie sich vor, Sie wohnen in einem Land, indem Steuerbehörde und Steuerzahler an einem Strang ziehen. Die Steuerbehörde fühlt sich verpflichtet, Unterstützung anzubieten. Die Steuerzahler nehmen ihrerseits die Steuerbehörde als Autorität wahr, die für das Wohl der Gemeinschaft arbeitet. Die Beziehung zwischen Steuerbehörde und Steuerzahler ist gekennzeichnet durch gemeinsame Verantwortung.   - *(Abkürzen, wenn C nicht als erstes Szenario)* Wenn Sie jetzt noch einmal an die Einflussmöglichkeiten denken, von denen wir vorher gesprochen haben, wobei Einfluss einerseits mittels Bestrafungen ausgeübt werden kann, andererseits aber auch durch Fachwissen und dem Geben von Informationen. Wenn Sie jetzt an die geschilderte Situation denken, übt die Steuerbehörde in dieser Situation Einfluss aus? Wenn ja, wie? (bzw. Warum nicht) *(Nur nachfragen, wenn nicht hypothesenkonform geantwortet wird)* - Wenn Sie jetzt noch einmal an die vorherigen Fragen zu Vertrauen denken: Jemand kann dem Gegenüber einerseits automatisch vertrauen, ohne darüber nachzudenken, weil der andere beispielsweise sympathisch ist, andererseits kann jemand dem Gegenüber auch vertrauen, weil er weiß, dass dieser kompetent ist und dieselben Ziele verfolgt. Vertrauen die Steuerzahler in dieser Situation der Steuerbehörde? Wenn ja, wie zeichnet sich das Vertrauen aus? (bzw. Warum nicht?) - Welche Ereignisse könnten vorgefallen sein, die zu dieser Veränderung in der Beziehung zwischen Steuerzahler und Steuerbehörde geführt haben? - Wenn Sie an Ihre Situation als Selbstständiger in Österreich denken, erinnern Sie sich an konkrete Ereignisse in letzter Zeit, von denen Sie sagen, das könnte/wird das Vertrauen in die Steuerbehörde beeinflussen?   ZU DEN ZWEI: Gibt es zu diesen Punkten noch Fragen von eurer Seite? | B. TAX – CLIMATE – INTERPLAY OF POWER AND TRUST  Now, I would like to describe some situations to you, which could depict the relationship between tax authority and taxpayer and afterwards, I am going to ask you some questions to it. I am kindly asking you now to imagine the following situations: *(Adjust the beginning if the scenarios, depending on which to start with)*  COUNTERBALANCE:   \| - A S C \| - S A C \| - C A S \| \| --- \| --- \| --- \| \| - A C S \| - S C A \| - C S A \|   Antagonistic Climate: (CP: ↑, LP: ↓, RBT: ↓, IT:0)  You live in a country, in which the tax authority and the taxpayer view themselves with suspicion. The tax authority views the taxpayers as criminals, who want to get rich and use every opportunity to evade taxes. On the other hand, the taxpayers feel persecuted and perceive the tax authority as hunters. The relationship between the tax authority and the taxpayer is marked by relentless and ruthless behaviour.   - If you may now think back to the possibilities to influence, which we talked about previously, whereas influence can be exerted via punishment, but also expertise and sharing information. If you now think of the depicted situation, does the tax authority exert its influence in this situation? If yes, how? (respectively: Why not?) *(only ask, if not answered consistently with the hypotheses)* - If you now think back to the questions about trust in institutions: on the one hand, a person can trust its counterpart automatically, on the other hand a person can also trust its counterpart, because the person knows, that the counterpart is competent and pursues the same goals. In this situation, do the taxpayers trust the tax authority? If yes, how can this trust be characterized? (respectively: why not?)   Change: Imagine now, that five years have passed and you still live in that country. Now, there have been some changes and the situation between taxpayers and the tax authority has been changed:    Service climate (CP: ↓, LP: ↑, RBT: ↑, IT: ↓)  You live in a country, where the taxpayers and tax authority work together on a basis of rules and standards and they behave like a company and their customers. The relationship between the tax authority and the taxpayer is characterized by the idea of a service.   - *(shorten, if S not as the first scenario)* If you now think again of the possibilities to influence, which we talked about previously, whereas influence is exerted by punishment, but also by expertise and sharing information. If you now think of the depicted situation, does the tax authority exert influence? If yes, how? (respectively: Why not?) *(only ask, if not answered consistently with the hypotheses)* - If you now think back to the questions about trust in institutions: on the one hand, a person can trust its counterpart automatically, without thinking about it, because for example the counterpart is likeable. On the other hand, a person can also trust its counterpart, because the person knows, that the counterpart is competent and pursues the same goals. If you now think of this situation, do the taxpayers trust the tax authority? If yes, how can this trust be characterized? (respectively: why not?) - Which incidence could have occurred, which led to the change in the relationship between the taxpayer and the tax authority?   Change: Imagine, another five years have passed and you still live in this country. Again, there have been changes and the situation between the taxpayers and the tax authority have been changed:  Confidence Climate: (CP: 0, LP: ↓, RBT: ↓, IT: ↑) Imagine, you live in a country, in which taxpayers and tax authority pull together. The tax authority feels obliged to provide support. The taxpayers for their part perceive the tax authority as an authority, working for the welfare of the community. The relationship between tax authority and taxpayer is characterised by shared responsibility.   - *(shorten, if C is first scenario)* If you now may think back to the possibilities to influence, which we talked about previously, whereas influence can be exerted by punishment, but also by expertise and sharing information. If you now think of the depicted situation, does the tax authority exert influence? If yes, how? (respectively: Why not?) *(only ask, if not answered consistently with the hypotheses)*      - If you now think back to the questions about trust in institutions: on the one hand, a person can trust its counterpart automatically, without thinking about it, because for example the counterpart is likeable. On the other hand, a person can also trust its counterpart, because the person knows, that the counterpart is competent and pursues the same goals. In this situation, do the taxpayers trust the tax authority? If yes, how can this trust be characterized? (respectively: why not?)      - Which incidence could have occurred, which led to the change in the relationship between the taxpayer and the tax authority? - If you think of your situation as a self-employed in Austria, do you remember specific events lately, you think could/will the trust in the tax authority be influenced?   REGARDING THOSE TWO: Are there any more questions on these topics from your side? |

| German Original | English Translation |
| --- | --- |
| 1. ENDE   *(Falls im Gespräch bis dahin nicht genannt wurde):*  Sie haben gar nicht erwähnt, ob bei Ihnen schon eine Steuerprüfung durchgeführt wurde:  **Wurde in Ihrem Unternehmen bereits eine Steuerprüfung durchgeführt?**   - Ja - Nein   Sie haben noch gar nicht erwähnt, in welcher Branche Sie tätig sind: **Branche**: ________  *(Wenn noch nicht gefragt:)*  **Beschäftigen Sie einen Steuerberater für Ihre Steuererklärung?**   - Ja - Nein   **Abschluss:**  Meiner Ansicht nach haben wir jetzt die wesentlichsten Ursachen für kooperatives Verhalten mit der Steuerbehörde besprochen - Möchten Sie vielleicht noch etwas hinzufügen oder fällt Ihnen spontan noch etwas ein, das wichtig ist und wir noch nicht erörtert haben?  Darf ich Sie bitten, in ein, zwei Sätzen den für Sie wichtigsten Aspekt dieses Interviews zusammenfassen?  Vielen Dank für Ihre Zeit und Ihre Hilfe.  Übersicht (schon Genanntes abhaken)  Auszahlungsbestätigung! | 1. END   *(In case it has not been mentioned so far):*  You have not mentioned yet, whether you have faced a tax audit already:  **Has there been a tax audit in your company already?**   - Yes - No   You have not mentioned yet, in which industry you work: **industry:** ________  *(if not asked yet:)*  **Do you employ a tax accountant for your tax audit?**   - Yes - No   **Conclusion:**  In my opinion, we have talked about the most important reasons for cooperative behaviour with the tax authority – Would you maybe like to add something or does something come to your mind, which is important that we have not talked about yet?  May I ask you to conclude in two sentences that aspect of the interview, which is most important for you?  Thank you for your time and your help.  Overview (mark the already mentioned)  Payment confirmation! |

| German Original | English Translation |
| --- | --- |
| **Jahresumsatz:**   - Weniger als € 25,000 - Zwischen € 25,000 und € 50,000 - Zwischen € 50,000 und € 100,000 - Zwischen € 100,000 und € 250,000 - Zwischen € 250,000 und € 500,000 - Zwischen € 500,00 und € 1,000,000 - Über € 1,000,000 | **Annual Sales:**   - less than € 25,000 - between € 25,000 und € 50,000 - between € 50,000 und € 100,000 - between € 100,000 und € 250,000 - between € 250,000 und € 500,000 - between € 500,00 und € 1,000,000 - more than € 1,000,000 |

| German Original | English Translation |
| --- | --- |
| Ein Unternehmen und die Unternehmensleitung kann mit Hilfe von Bestrafungen Einfluss auf seine Mitarbeiter nehmen, indem es Druck ausübt, seine Mitarbeiter kontrolliert und mit Sanktionen droht, wie beispielsweise einer Versetzung, Kündigung oder Nichtauszahlung eines Bonus. | A company and the management can exert influence on their employees by applying pressure, control their employees and threaten to impose sanctions, for example a relocation, termination or not paying bonus. |

| German Original | English Translation |
| --- | --- |
| Ein Unternehmen und die Unternehmensleitung haben die Möglichkeit, mit Hilfe von Belohnungen Einfluss auf seine Mitarbeiter zu nehmen. So sind etwa eine Gehaltserhöhung, aber auch Lob und wertschätzende Worte Einflussmöglichkeiten, ebenso wie das Abwenden des Verlusts des Arbeitsplatzes oder die Eröffnung von Aufstiegschancen. | A company and the management have the possibility to influence their employees with the help of reward. A salary increase, but also praise and appreciative words are possibilities to influence, also averting the loss of the job or disclosing to opportunity for promotion. |

| German Original | English Translation |
| --- | --- |
| Das Unternehmen und seine Leitung hat Einfluss auf die Mitarbeiter aufgrund der rechtmäßigen Position des Unternehmens gegenüber den Mitarbeitern, die die Stellung des Unternehmens nicht in Frage stellen. | The company and its management can influence their employees due to its legal position towards employees, who do not question the company´s position. |

| German Original | English Translation |
| --- | --- |
| Ein Unternehmen und die Unternehmensleitung hat die Möglichkeit Einfluss auf seine Mitarbeiter zu nehmen, wenn diese sich mit dem Unternehmen identifizieren und ihm gegenüber das Gefühl von Verbundenheit und Wertschätzung empfinden. | A company and its management have the possibility to influence their employees when they identify with the company and have the feeling of affinity and appreciation towards it. |

| German Original | English Translation |
| --- | --- |
| Ein Unternehmen und die Unternehmensleitung hat Einfluss auf seine Mitarbeiter, da die Unternehmensleitung Experten auf ihrem Gebiet sind. | A company and the management have the possibility, because the management are experts on their field. |

| German Original | English Translation |
| --- | --- |
| Eine Einflussmöglichkeit des Unternehmens kann darin bestehen, dass das Unternehmen Informationen weitergibt, welche die Mitarbeiter dazu veranlassen, ihre Aufgaben im Sinne des Unternehmens zu erledigen. | One possibility of the company to influence can be, that the company shares information, which causes the employees to handle their tasks in the interest of the company. |

| German Original | English Translation |
| --- | --- |
| Sie wohnen in einem Land, indem sich Steuerbehörde und Steuerzahler gegenseitig argwöhnisch beobachten. Die Steuerzahler werden von der Steuerbehörde als Verbrecher gesehen, die sich jederzeit nur bereichern wollen und jede Möglichkeit nutzen, Steuern zu hinterziehen. Die Steuerzahler fühlen sich hingegen verfolgt und nehmen die Steuerbehörde als Jäger wahr. Die Beziehung zwischen Steuerbehörde und Steuerzahler ist gekennzeichnet durch unbarmherziges und rücksichtsloses Verhalten. | You live in a country, in which the tax authority and the taxpayer view themselves with suspicion. The tax authority views the taxpayers as criminals, who want to get rich and use every opportunity to evade taxes. On the other hand, the taxpayers feel persecuted and perceive the tax authority as hunters. The relationship between the tax authority and the taxpayer is marked by relentless and ruthless behaviour. |

| German Original | English Translation |
| --- | --- |
| Sie wohnen in einem Land, indem Steuerbehörde und Steuerzahler auf Basis von Regeln und Standards zusammenarbeiten und sich wie ein Unternehmen und seine Kunden verhalten. Die Beziehung zwischen Steuerbehörde und Steuerzahler ist vom Servicegedanken geprägt. | You live in a country, where the taxpayers and tax authority work together on a basis of rules and standards and they behave like a company and their customers. The relationship between the tax authority and the taxpayer is characterized by the idea of a service. |

| German Original | English Translation |
| --- | --- |
| Stellen Sie sich vor, Sie wohnen in einem Land, indem Steuerbehörde und Steuerzahler an einem Strang ziehen. Die Steuerbehörde fühlt sich verpflichtet, Unterstützung anzubieten. Die Steuerzahler nehmen ihrerseits die Steuerbehörde als Autorität wahr, die für das Wohl der Gemeinschaft arbeitet. Die Beziehung zwischen Steuerbehörde und Steuerzahler ist gekennzeichnet durch gemeinsame Verantwortung. | Imagine, you live in a country, in which taxpayers and tax authority pull together. The tax authority feels obliged to provide support. The taxpayers for their part perceive the tax authority as an authority, working for the welfare of the community. The relationship between tax authority and taxpayer is characterised by shared responsibility. |

**Interview guideline for tax auditors**

| German Original | English translation |
| --- | --- |
| **Informationen zum Interview**  Wir führen im Rahmen zweier Diplomarbeiten an der Universität Wien Interviews durch und untersuchen die Strategien von SteuerbeamtInnen im Umgang mit SteuerzahlerInnen innerhalb einer Steuerprüfung.  Unser Gespräch wird mit einem Diktiergerät aufgezeichnet, aber Ihre Daten werden selbstverständlich anonym und vertraulich behandelt. Das gesamte Gespräch wird gleich nach der Transkription wieder gelöscht.  Wichtig ist es zu erwähnen, dass es keine richtigen und falschen Antworten gibt. Antworten Sie einfach aus dem Bauch heraus.  Wir freuen uns an Ihren Erfahrungen teilhaben zu können und bedanken uns herzlich, dass Sie sich Zeit für uns genommen haben.  Dauer: max. 2h, wahrscheinlich kürzer  Wir bitten Sie dafür zu sorgen, dass wir während des Interviews nicht unterbrochen werden, bitte schalten Sie ggf. Ihr Handy aus.  Wir starten zunächst mit ein paar kurzen Fragen zu Ihrer Person und Ihrer Tätigkeit und stellen im weiteren Verlauf Fragen zu Ihren Strategien im Umgang mit den SteuerzahlerInnen. | **Information on the interview**  In the context oft two diploma theses at the University of Vienna we are conducting interviews in order to investigate the strategies of tax officials dealing with taxpayers within a tax audit.  Our conversation is being recorded with a voice recorder, but of course your data will be treated anonymously and confidentially. The whole conversation will be deleted right after it has been transcribed.  Please note: there are no right or wrong answers. Just answer intuitively.  We are happy you are sharing your experiences with us and would like to thank you cordially for taking your time for us.  Duration: max. 2h, probably shorter  We kindly ask you to ensure that we will not be interrupted during the course of the interview, if necessary, please turn off your phone.  We are going to start with a few questions regarding your person and your job and will proceed to ask questions concerning your strategies when dealing with taxpayers. |

| German Original | English translation |
| --- | --- |
| **Kurzfragebogen**  **Geschlecht:** männlich/weiblich  **Wie lange sind Sie schon als SteuerprüferIn tätig (in Jahren)?**  **Welche Art von Prüfungen führen Sie durch?** *Offen fragen! Nicht alles vorlesen!*   - Prüfungen in Großbetrieben - Prüfungen in mittelgroßen Betrieben - Prüfungen in Kleinbetrieben - Keine Prüfungen in Unternehmen - LeiterIn einer Abteilung für Prüfungen, führe selbst keine Prüfungen durch   **Wie viele Prüfungen führen Sie durchschnittlich im Jahr durch?**  **Wie lange dauert durchschnittlich eine Prüfung an?**  **Wie alt sind Sie?**  Legende (für den Interviewer):  (I) implizites Vertrauen  (R) rationales Vertrauen  (C) coercive Macht  (L) legitime Macht  Rote und kursive Schrift: Hinweise für den Interviewer: Sachen, die Befragter erwähnt hat, sollen aufgegriffen werden.  Schwarze und kursive Schrift: was wir zusätzlich erwähnen können | **Short questionnaire**  **Gender:** male/female  **How long have you been working as a tax auditor (duration in years)?**  **Which kind of audits are you conducting?** (Open question! Do not read everything aloud!)   - Audits in large companies - Audits in medium-sized companies - Audits in small companies - No audits in companies - Department head, does not conduct audits himself   **How many audits do you conduct annually on average ?**  **How long does an average audit take?**  **How old are you?**  Legend (for the interviewer):  (I) implicit trust  (R) rational trust  (C) coercive power  (L) legitimate power  Red and italic text: Advice for the interviewer: Things mentioned by the interviewee should be implemented into the interview.  Black and italic: could be mentioned additionally |

| German Original | English translation |
| --- | --- |
| 1. Einstiegsfrage  - Wie sieht eine Steuerprüfung üblicherweise bei Ihnen aus? (In 4 bis 5 Eckpunkten)   - Was sind denn Ihre Aufgaben bei einer Steuerprüfung? - Was ist Ihr Ziel einer Prüfung? *(Geht es um korrektes Zahlen/korrektes Abführen der Steuer* ***jetzt*** *oder auch für die* ***Zukunft****?)* - (C, L, R, I) **Mal angenommen, Sie wollen etwas vom Steuerzahler *(z.B. wichtige fehlende Unterlagen/dass er die Nachzahlung fristgerecht bezahlt/dass er sich im Abschlussgespräch kooperativ verhält)*. Wie gehen Sie dann vor?**   - **Was könnte man noch machen?** Was fällt Ihnen noch ein? Welche Möglichkeiten setzen Sie noch ein? Was ziehen Sie noch in Betracht? - Sie haben gerade gesagt, dass Sie drohen/Druck ausüben/…. *(die von ihm genannten Methoden aufzählen)*, um das zu bekommen, was Sie vom Steuerzahler wollen *(z.B. wichtige fehlende Unterlagen/rechtzeitig/Ortauswahl/fristgerechte Nachzahlung)*. Wie reagiert dann der Steuerzahler darauf/auf …? - Gibt es noch andere Methoden, die Sie einsetzen? | 1. Introductory questions  - How does one of your tax audits usually look like? (4 to 5 key points)   - What are your tasks during a tax audit? - What is your goal with a tax audit? (*Is it only about correct tax payments* ***now*** *or also* ***in the future****?*) - (C, L, R, I) **Assuming you want something from the taxpayer *(e.g. important missing documents/ him to pay subsequent payments on time/ him to cooperate during the concluding conversation)*: How will your approach look like?**   - **What else could one do?** What else comes to your mind? Which other options do you see? Which other options do you consider? - You just said you would be threatening/ apply pressure/ … *(list the methods mentioned by the interviewee)*, to get what you want from the taxpayer (*e.g. important missing documents/on time/ choice of location/timely payment*). How will the taxpayer then react to …? - Are there any other methods you employ? |

| German Original | English translation | |
| --- | --- | --- |
| 1. Macht auf Verhalten  - (C) Wenn der Klient, wie vorhin beschrieben, nicht kooperiert und Sie dann mehr Druck ausüben/Strafen androhen/…. machen.   - Wie reagiert dann der Klient darauf? - (C) Wenn der Klient, wie vorhin beschrieben, nicht kooperiert und Sie dann Vergünstigungen/Erleichterungen/Entgegenkommen/Belohnungen anbieten.   - Wie reagiert *(z.B. beim Fehlen von Unterlagen/Abschlussgespräch)* dann der Klient typischerweise darauf? - (L) Wie wirkt es sich aus *(z.B. auf das korrekte Vorlegen der Unterlagen/Nachzahlungen/ usw.)*, wenn Sie den Leuten Infos geben/Tipps geben/unterstützen/beraten/erklären?   - Wie reagiert dann der Klient darauf? - (L) Welche Rolle spielt ihr Auftreten als Vertreter des Staates *(im Umgang mit dem Steuerzahler/beim korrekten Vorlegen der Unterlagen, …)*?   - Wie reagiert dann der Klient darauf? - (L) Nehmen wir an, Sie haben einen Klienten, der prinzipiell vom Steuersystem überzeugt ist und Ihre Tätigkeit als wichtig empfindet. Wie reagiert der Klient darauf, wenn Sie etwas von ihm wollen *(fehlende Unterlagen/Abschlussgespräch}*?   - Wie kann ich mir das anhand eines Beispiels konkret vorstellen? - (L+C) Sie haben jetzt angeführt, dass man mit … *(die erwähnten Methoden aufgreifen)* *(z.B. Überzeugungskraft/dem eigenen Fachwissen/…)* arbeiten kann und aber auch mit … *(z.B. dem Androhen von Konsequenzen/mehr Druck/…).*   - Wo sind denn da die Unterschiede im Ergebnis *(Ort festlegen, Unterlagen rechtzeitig/vollständig, Schlussbesprechung, Nachzahlung)*? Wie unterscheidet sich das? | 1. Power on trust  - (C) If the client, as described before, does not cooperate and you then pressure him/threaten punishment/ (…).   - How will the client then react? - (C) If the client, as just described, does not cooperate and you offer benefits/facilitations/goodwill/rewards.   - How will the client (*e.g. in the case of missing documents or during a concluding conversation*) then react typically? - (L) How does it affect (*e.g. the correct submission of documents/subsequent payments/ etc.*), if you give the people information/give them advice/support them/explain things?   - How will the client react to this? - (L) Which role does your demeanour as a representative of the state (*during the interaction with the taxpayer/during the correct submission of documents, …*) play?   - How will the client then react to this? - (L) Assuming you have a client who is generally convinced of the tax system and considers your occupation to be important. How does the client react, if you want something from him *(missing documents/concluding conversation)*?   - How can I imagine that given a concrete example? - (L+C) You just stated that one can work by (quote the mentioned methods) *(e.g. persuasiveness/own specific knowledge/ …)* but also by *(e.g. threatening punishment/more pressure/…)*.   - Where exactly are the differences concerning the final result *(determine location, documents on time/complece, subsequent payments)*? How does it differ? |  |

| German Original | English translation |
| --- | --- |
| 1. Vertrauen auf Verhalten  - Sie haben jetzt … gesagt/angeführt/aufgezählt. Jetzt mal eine andere Frage: Welche Rolle spielt Vertrauen in Sie und in die Behörde in der Prüfungssituation?   - Wie sieht man denn das Vertrauen? Wie macht es sich bemerkbar? An welchen Reaktionen sieht man Vertrauen? - Warum vertrauen Sie Steuerzahlern: Wann merken Sie, der oder die ist vertrauenswürdig? - Warum nehmen Sie an, dass die Steuerzahler Ihnen vertrauen?      - (R) Welche Gründe haben sie Ihnen zu vertrauen?   - Und wie wirkt sich dies *(nur die genannten Gründe aufzählen)* auf das Verhalten *(bei Nachzahlungen/fehlende Unterlagen/im Abschlussgespräch)* aus?     - Z.B. Abschlussbesprechung, ist es da anders (die Gründe) als beim Einfordern von fehlenden Unterlagen?   - Können Sie da was machen, dass der Steuerzahler tatsächlich das akzeptiert und wirklich fristgerecht überweist?      - (R) Ich stelle mir vor, dass Sie gerne zügig die Prüfung beenden wollen und der Steuerzahler mit keiner/einer möglichst geringen Nachzahlung rausgehen will. Irgendwie befinden Sie sich ja da in einer gegenseitigen Abhängigkeit.   - Welche Auswirkung kann diese Abhängigkeit auf das Verhalten der Steuerzahler haben? - (R) Sagen wir, manche Steuerzahler finden es wichtig, Steuern für staatliche Leistungen zu zahlen *(z.B. neue Krankenhäuser, neue Straßen*). Welche Rolle spielt diese Einstellung beim Vertrauen Ihnen gegenüber?   - Und wie wirkt sich das auf das Verhalten in der Prüfungssituation aus? - (R) Was macht ihr, damit die Steuerzahler das Gefühl haben, dass ihr es nicht böse mit ihnen meint/dass ihr euch gut auskennt/dass ihr engagiert rüberkommt?   - Wie verhält sich der Steuerzahler dann? - (R) Wie sieht es mit Medienberichten über Steuerhinterziehung aus? Wie wirkt sich so etwas auf das Vertrauen in *(ins Steuersystem)* Sie in der Prüfungssituation aus? - (I) Welche Rolle spielt Sympathie? Wie wirkt die sich auf das Vertrauen in Sie aus?   - Wie reagieren die Steuerzahler dann? Wie verhalten sich die Steuerzahler dann konkret?   - Wie entsteht Sympathie?/Wie erreichen Sie Sympathie?/Was machen Sie, um sympathisch rüberzukommen? - (I) Welchen Einfluss hat es, wenn die Steuerzahler das Gefühl haben, sie beide können offen über alles reden, bzw. dass Sie beide auf derselben Wellenlänge sind?   - Wie wirkt das auf das Verhalten der Steuerzahler aus?   - Wie machen Sie das, diese gemeinsame Wellenlänge zu erreichen? - (I) Welche Auswirkungen haben die Erfahrungen *(sowohl positive als auch negative Erfahrungen und deren Auswirkungen abfragen)*, die die Steuerzahler bis jetzt mit der Steuerbehörde gemacht haben, auf das Vertrauen?   - Welche Auswirkung kann das auf die Reaktionen der Steuerzahler in der Prüfungssituation haben? - Wir haben jetzt über verschiedene Ursachen/Gründe wie z.B. … *(aufzählen, was die Person erwähnt hat)* gesprochen, warum Steuerzahler Ihnen vertrauen. Gibt es da Unterschiede im Ergebnis *(z.B. bei der Schlussbesprechung, Aushändigen fehlender Unterlagen)*? | C. Trust on behaviour   - You just said/state/listed … . Now a different question: Which role does trust in you and the authority in the audit situation play?   - How does one see the trust? How is it perceivable? Which reactions show signs of trust? - Why do you trust the taxpayer? When do you notice that he or she is trustworthy? - Why do you assume the taxpayers trust you? - (R) Which reasons do they have to trust you?   - And how does this *(now list the reasons stated)* affect the behaviour *(concerning subsequent payments/missing documents/during the concluding conversation)*?     - E.g. the concluding conversation: is it different (the reasons) than when you demand missing documents   - Can you somehow influence that the taxpayer actually accepts this and transfers the money on time? - (R) I imagine that you want to end the audit quickly and that the taxpayer wants to leave with no/a minimal subsequent payment. Somehow this creates a mutual dependency.   - Which consequences can this dependency have on the behaviours of the taxpayer? - (R) Let’s say some taxpayers consider it important to pay taxes for public services *(e.g. new hospitals, new streets)*. Which role does this attitude play concerning the trust in you?   - And how does this affect the behaviour in an audit situation? - (R) What do you do in order to give the taxpayer the feeling that you mean no harm/are well informed/make a committed impression?   - How does the taxpayer then behave? - (R) How about reports in the media about tax evasion? How does something like this affect the trust in you *(in the tax system)* in the audit situation? - (I) Which role does sympathy play?  How does it affect the trust in you?   - How do the taxpayers react then?   - How does sympathy arise? How do you achieve sympathy? What do you do to act likeable? - (I) Which effect does it have, when the taxpayer is under the impression that you both can talk freely about everything, or else that you are both on the same wavelength?   - How does this affect the behaviours of the taxpayer?   - How do you do this, reach the same wavelength? - (I) Which effects do the experiences *(ask for both positive and negative experiences and their consequences)* the taxpayer had so far with the tax authorities on the trust?   - Which impact can this have on the taxpayer´s reactions in a audit situation? - We have now talked about different causes and reasons like for example (quote what the person has said), why the taxpayer trust you. Are there differences concerning the result *(e.g. during the concluding conversation, delivering missing documents)*? |

| German Original | English translation |
| --- | --- |
| D. Wechselspiel von Macht und Vertrauen   - Welchen Einfluss haben Ihre Beeinflussungsstrategien/Methoden wie … *(aufzählen, was die Person erwähnt hat)* auf das Ihnen entgegengebrachte Vertrauen? - Wie wirkt sich das Vertrauen, das Ihnen entgegengebracht wird, auf die Beeinflussungsstrategien wie … *(aufzählen, was die Person erwähnt hat)* aus?   **Legitime Macht auf Vertrauen**  Sie haben erzählt, dass Sie durch gewisse Strategien wie … *(aufzählen, was die Person erwähnt hat)* *(z.B. ihre Expertise/ Position/gesellschaftliche Ziele des Steuerzahlens/Überzeugungskraft)* die Leute dazu bringen gut mitzuarbeiten.   - (R) Wie wirken sich diese *(Strategien)* *(aufzählen, was die Person erwähnt hat)* darauf aus, wie kompetent/wohlwollend/motiviert Sie wahrgenommen werden?   - In welcher Weise verändert dies *(die Strategien)* das Vertrauen in Sie? - (I) Wie wirkt sich das … *(aufzählen, was die Person erwähnt hat)* darauf aus, wie sympathisch oder verständnisvoll Sie wahrgenommen werden?   - In welcher Weise verändert das das Vertrauen in Sie?   **Coercive Macht auf Vertrauen**  Sie haben erzählt, dass Sie in manchen Situationen … *(erzählte Situationen aufgreifen –Machtsituationen)* *(z.B. Druck, Strafen, Kontrollen, Anbieten von Vergünstigungen)* einsetzen.   - Wie wirkt sich das auf das Vertrauen aus? - (R) Wie wirkt es sich auf das Vertrauen aus, wenn Sie eine Person bestrafen/eine Nachzahlung verlangen/belohnen, wenn die Person eigentlich das Steuersystem gut findet? - (R) Wie wirkt es sich auf das Vertrauen aus, wenn Sie eine Person bestrafen/eine Nachzahlung verlangen/belohnen, wenn die Person Sie als sehr kompetent wahrnimmt? - (R) Wie wirkt es sich auf das Vertrauen aus, wenn Sie eine Person bestrafen/eine Nachzahlung verlangen/belohnen, wenn die Person Sie aber als sehr motiviert und engagiert wahrnimmt? - (I) Wie wirken sich Strafen/Nachzahlungen/Belohnungen etc. darauf aus, wie sympathisch oder verständnisvoll Sie wahrgenommen werden?   **Rationales Vertrauen auf Macht**   - (L) Nehmen wir mal an, Ihnen wird vom Steuerzahler Vertrauen entgegengebracht *(dass Sie sowieso alles korrekt machen)*. Wie werden Sie dann wahrgenommen *(als Experte, Vertreter des Staates, respektieren sie Ihre Position, etc.)*? - (C) Jetzt gehen wir mal davon aus, dass die Leute Ihnen vertrauen, weil Sie als engagiert/wohlwollend/hilfsbereit wahrgenommen werden. Wie wirkt sich das dann aus, wenn Sie eine Nachzahlung einfordern?   **Implizites Vertrauen auf Macht**   - Wenn die Leute Sie sympathisch finden und Ihnen deshalb vertrauen: Was machen Sie dann, wenn z.B. Unterlagen fehlen/es um die Verhandlung von Nachzahlungen geht? - (L) Sie sind mit dem Steuerzahler auf einer Wellenlänge. Sie finden sich sympathisch/er ist auch ein Mann/Sie beide haben die gleichen Hobbys… Werden Sie dann eher als unterstützend wahrgenommen *(z.B. ja, das macht der schon, der kennt sich)*? - (C) Sie sind mit dem Steuerzahler auf einer Wellenlänge. Sie finden sich sympathisch. Wie wirkt sich das darauf aus, wenn Sie Strafen androhen/Vergünstigungen anbieten etc.? | 1. Interplay of power and trust  - Which impact do your influencing strategies/methods like … *(list what the person mentioned)* have on the trust you are met with? - How does the trust you are met with affect the influencing strategies like … *(list what the person mentioned)*?   **Legitimate power on trust**  You explained that, by employing strategies like *(list what the person mentioned) (e.g. your expertise/position/societal goals of tax paying/persuasiveness)*, you get people to cooperate.   - (R) How do these *(strategies)* *(list what the person mentioned)* affect how competent/favourable/motivated you are being perceived?   - How does this *(the strategies)* change the trust in you? - (I) How does this … *(list what the person mentioned)* affect, how likeable or understanding you are being perceived?   - How does this change the trust in you?   **Coercive power on trust**  You said that in certain situations … *(refer to the mentioned situations – situations of power)* you employ *(e.g. pressure, punishment, controls, offering benefits).*   - How does this affect the trust? - (R) How does it influence the trust, when you punish a person/demand a subsequent payment/reward, if the person actually likes the tax system? - (R) How does it influence the trust, when you punish a person/demand a subsequent payment/reward, if the person perceives you as very competent? - (R) How does it influence the trust, when you punish a person/demand a subsequent payment/reward, if the person perceives you as very motivated and committed? - (I) How do punishments/subsequent payments/rewards (…) affect, how likeable or understanding you are being perceived?   **Rational trust on power**   - (L) Let’s assume you are met with trust by the taxpayer *(that you are doing everything correctly).* How will you be perceived *(as an expert, representative of the state, do they respect your position, etc.)*? - (C) Now let’s assume people trust you, because you are perceived as committed/favourable/helpful. Which effect does this produce, when you demand a subsequent payment?   **Implicit trust on power**   - If people perceive you as likeable and thus trust you: What do you do then, if for example documents are missing/the subsequent payment is being negotiated? - (L) You and the taxpayer are on the same wavelength. You like each other/he is also male/you have the same hobbies….   Are you then perceived as being more supportive *(e.g. yes, he will do that, he knows himself)*?   - (C) You and the taxpayer are on the same wavelength. You like each other. How does this affect, which punishments you threaten/benefits you offer etc.? |

| German Original | English translation |
| --- | --- |
| **Abschluss:**  Meiner Ansicht nach haben wir jetzt die wesentlichsten Aspekte besprochen - Möchten Sie vielleicht noch etwas hinzufügen oder fällt Ihnen spontan noch etwas ein, das wichtig ist und wir noch nicht erörtert haben?  Wiederholen um was es ging und dann: Darf ich Sie bitten in einem Satz den für Sie wichtigsten Aspekt dieses Interviews zusammenfassen?  Wir führen momentan noch Probeinterviews durch. Haben Sie vielleicht noch Vorschläge für Verbesserungen/Veränderungen? Gab es Fragen, die nicht gut beantworten werden konnten oder schwierig waren?  Vielen Dank für Ihre Zeit und Ihre Hilfe. | **Conclusion:**  In my opinion we talked about the most important aspects.  Would you maybe like to add something else? Does something come to your mind you would consider important that we have not talked about yet?  Repeat what this was about and then:  May I ask you to conclude the most important aspects of this interview in a single sentence?  We are currently still conducting the pretest interviews. Do you maybe have suggestions for improvements/changes? Are there questions you considered too hard to answer or to understand?  Thank you for your time and help. |

Transcripts of all interviews

See: <https://osf.io/nv285/>

Example of a measurement instrument based on the current findings

Based on the current results we developed new scales and adapted existing questionnaires (i.e., Hofmann et al., 2014) on power, trust and tax compliance This questionnaire consisted of the following scales: coercive power (3 items), setting deadlines (5 items), reward power (3 items), legitimate power (16 items), information power (4 items), expert power (4 items), referent power (4 items), justice (5 items), transparency (5 items), participation (5 items); trust in the tax authorities assessed as implicit trust (3 items), personal support (5 items), reason-based trust (7 items) and respect (5 items); tax behavior was assessed as tax honesty (4 items), tax avoidance (4 items), tax evasion (4 items), accepting (5 items) as well as stalling tax behavior (5 items) and additional scales on perceived climate and motives for cooperation, not relevant for the current study. All items were answered on 7-point Likert-scales ranging from 1 = totally disagree to 7 = totally agree or from 1 = very unlikely to 7 = very likely.

Via a market-research agency, we presented this instrument to a sample of 100 Austrian self-employed taxpayers, of which two were excluded s they had never filed in a tax return, resulting in a final sample of 98 self-employed taxpayers (62.2% men, Mage = 49.39, SDage = 10.13). Almost all self-employed (98.0%) reported to have at least some experience with the tax authority and the majority (74.5%) engaged a tax advisor. Most of them reported a turnover of less than 25.000 Euro (40.8%; 25.001 Euro – 50.000 Euro: 21.4%; 50.000 Euro – 100.000 Euro: 14.3%; 100.001 – 250.000 Euro: 15.3%; more than 250.001 Euro: 8.1%) and to employ no personnel (69.4%; 1employee: 14.3%; 2-10 employees: 13.2%; more than 10 employees: 3.0%). Participants were paid 1.50 EUR (approximately 1.60 USD at the time) for participation

Based on the collected data, we conducted explorative principal component factor (PCA) analysis with varimax rotation to examine whether the new categories belong to the expected theoretical categories. Results showed that the items of coercive power, reward power, reason based trust, implicit trust, tax honesty, tax evasion, tax avoidance, accepting and stall tax behavior form one factor. Only the factor analysis for legitimate power indicated that the different categories of legitimate power also load on different respective factors. However, an analyses in which three items with loadings smaller than .40 were deleted showed that all other items can be forced to load on one factor which indicates that the different categories of legitimate power are highly related and share a common variance. All scales had Cronbach alpha’s equal or higher than .77. Items, factor loadings and Cronbach alpha’s can be seen in Table A1 and A2. Correlations between all scales are presented in Table A3.

This survey study is only a first attempt to examine the factor structure and relations of the determinants of the tax relationship and tax behavior. When interpreting the results, the small sample size and the cross-sectional design should be considered as limitations.

**Table A1** Items of power and trust scales, results of the PCA, rotated factor loadings, and Cronbach alpha’s

| Scale | Item | FL | **α** |
| --- | --- | --- | --- |
| **Coercive Power** | Please read the following statements carefully. The statements refer to the Austrian tax authorities |  | .92 |
|  | The tax authority punishes severely. | .800 |  |
|  | The tax authority enforces its demands with audits and fines. | .829 |  |
|  | The tax authority prosecutes taxpayers with audits and fines. | .826 |  |
|  |  |  |  |
| **Deadlines** |  |  |  |
|  | The tax authority sets strict deadlines. | .832 |  |
|  | The tax authority resolutely demands compliance with deadlines. | .852 |  |
|  | The tax authority is uncompromising and tough when setting deadlines. | .824 |  |
|  | The tax authority has strict deadlines for documents. | .841 |  |
|  | The tax authority does not negotiate over deadlines. | .457 |  |
| **Reward Power** |  |  | .77 |
|  | The tax authority grants taxpayers concessions in a number of ways. | .880 |  |
|  | The tax authority grants taxpayers tax reliefs. | .625 |  |
|  | The tax authority rewards taxpayers in many ways. | .687 |  |
| **Legitimate Power** |  |  | .96 |
| **Legitimate Position Power** | The tax authority levies taxes based on legal guidelines. | .427 |  |
|  | Due to the legal situation, the tax authority is in the position to levy taxes. | -* |  |
|  | The tax authority is the legal representative of the State concerning tax collection. | -* |  |
|  | The tax authority can only work well, if the taxpayers submit their documents in order and on schedule. | .636 |  |
|  | In order to work efficiently, the tax authority depends on the taxpayers who fill in their tax forms correctly. | .605 |  |
|  | The tax authority cannot work well without the carefully edited documents of taxpayers. | .515 |  |
|  | If the tax authority had to wait for documents of taxpayers in the past, they must receive them quickly in the future. | .729 |  |
|  | The tax authority is an institution, taxpayers owe cooperation to because the tax authorities were fair in its past dealings. | .707 |  |
|  | The tax authority is an institution that can count on the support of taxpayers, because the authority supported taxpayers in the past. | .604 |  |
|  | The tax authority is an institution whose requests are matched by the taxpayers, because the authority has already undertaken a lot for the individual. | .656 |  |
|  | In order to be able to work, the tax authority depends on the taxpayers to fill in their tax forms carefully. | .497 |  |
|  | The tax authority is an institution, the taxpayers should unconditionally cooperate with, if taxpayers were disorganised in delivering their documents in the past. | .648 |  |
|  | The tax authority should especially receive diligently filled in tax returns from taxpayers whose tax returns were too often completed incorrectly in the past. | .474 |  |
|  | The tax authority is an institution taxpayers feel obliged to cooperate with, because of the good services for the taxpayers in the past. | .741 |  |
|  | The tax authority should especially obtain the tax return on schedule, if the tax return was handed in late in the past. | .695 |  |
|  | The tax authority has the right to levy taxes from taxpayers because of its position in the State. | -* |  |
| **Information Power** |  |  |  |
|  | The tax authority makes all taxpayers understand, which taxes they have to pay and to what extent they have to pay them. | .621 |  |
|  | The tax authority informs taxpayers of possible errors which might occur in their tax return. | .582 |  |
|  | The tax authority shares information understandably. | .490 |  |
|  | The tax authority explains regulations. | .684 |  |
| **Expert Power** |  |  |  |
|  | The tax authority knows how the correct tax return of each taxpayer has to look like. | -* |  |
|  | The tax authority is an expert on tax regulations and their implementation. | .632 |  |
|  | The tax authority knows how a correctly filled in tax return should look like. | .549 |  |
|  | The tax authority knows best regarding regulations related to taxes. | .596 |  |
| **Referent Power** |  |  |  |
|  | The tax authority is appreciated for their services by taxpayers. | .531 |  |
|  | The tax authority is acknowledged for their work by taxpayers. | .423 |  |
|  | The tax authority is respected by taxpayers. | .555 |  |
|  | The tax authority is regarded for their work by the taxpayers. | .525 |  |
| **Justice** |  |  |  |
|  | The tax authority treats all taxpayers equally. | .799 |  |
|  | The tax authority ensures that procedures for all taxpayers are fair. | .734 |  |
|  | The tax authority ensures that all taxpayers fulfil their obligations. | .575 |  |
|  | The tax authority ensures that all taxpayers are treated fairly. | .821 |  |
|  | The tax authority ensures that all taxpayers pay their fair share. | .768 |  |
| **Transparency** |  |  |  |
|  | The tax authority discloses the reasons for their decisions. | .656 |  |
|  | The tax authority justifies their decision-making processes in a comprehensible way. | .623 |  |
|  | The tax authority designs all processes and decisions in a transparent and comprehensible way. | .787 |  |
|  | The tax authority emphasizes transparency. | .779 |  |
|  | The tax authority designs procedures open and transparent. | .784 |  |
| **Participation** |  |  |  |
|  | The tax authority enables taxpayers to be involved in the employment of public money. | .461 |  |
|  | The tax authority allows taxpayers the possibility of participation. | .573 |  |
|  | The tax authority gives taxpayers the possibility to take part in the decision of how to use the tax money. | -* |  |
|  | The tax authority allows taxpayers to get involved in the improvement of procedures. | .666 |  |
|  | The tax authority allows taxpayers to bring in ideas for improvement. | .547 |  |
| **Implicit Trust** |  |  | .92 |
|  | I trust the tax authority usually without thinking about it. | .698 |  |
|  | I trust the tax authority usually without dealing with it deeply. | .684 |  |
|  | I trust the tax authority most of the time automatically. | .767 |  |
| **Personal Support** |  |  |  |
|  | I trust that, regarding my concerns, I will receive personal support at the tax authority. | .831 |  |
|  | I trust the people in the tax authority. | .872 |  |
|  | I trust that I will receive individual support of the tax authority. | .837 |  |
|  | I trust that concerns will be solved humanely by the tax authority. | .775 |  |
|  | I trust that someone in the tax authority is personally responsible for my problems. | .704 |  |
| **Reason Based Trust** |  |  | .96 |
|  | I trust the tax authority because there is no alternative. | .486 |  |
|  | I trust the tax authority because its decisions are politically supported. | .669 |  |
|  | I trust the tax authority because it is fulfilling its tasks well. | .851 |  |
|  | I trust the tax authority because it acts benevolently towards taxpayers. | .880 |  |
|  | I trust the tax authority because its goals seem plausible. | .772 |  |
|  | I trust the tax authority because it has committed employees. | .817 |  |
|  | I trust the tax authority because the economic stability guarantees that the tax authority can work. | .679 |  |
| **Respect** |  |  |  |
|  | I trust the tax authority, because my individual concerns are respected. | .861 |  |
|  | I trust the tax authority, because they respect me. | .921 |  |
|  | I trust the tax authority, because they respect taxpayers. | .923 |  |
|  | I trust the tax authority because respect towards the taxpayers is important for the officials. | .917 |  |
|  | I trust the tax authority, because I am treated respectfully. | .935 |  |

*Note:* Items are translations from the original German items. Results for legitimated factor represent the fixed solution.

**Table A2.** Items of tax compliance behavior scales and results of the exploratory factor analysis, rotated factor loadings, and Cronbach alpha’s

| Scale | | Item (English) | |  | |  | | **α** | |
| --- | --- | --- | --- | --- | --- | --- | --- | --- | --- |
| **Tax Honesty** |  | |  | |  | | .94 | |  |
|  | I pay my taxes honestly. | |  | | .933 | |  | |  |
|  | I try to provide all my tax documents correctly. | |  | | .901 | |  | |  |
|  | I am completely honest with the tax authority, concerning my taxes. | |  | | .963 | |  | |  |
|  | I am an honest taxpayer. | |  | | .915 | |  | |  |
| **Tax Evasion** |  | |  | |  | | .91 | |  |
|  | How likely would you conceal extra income of the tax authority, in order to reduce your tax burden? | |  | | .901 | |  | |  |
|  | How likely would you offer goods and services without a bill and conceal its profits in the tax records? | |  | | .900 | |  | |  |
|  | How likely would you specify bills as “business meals” in the tax documents, although it was a meal with friends? | |  | | .885 | |  | |  |
|  | How likely would you link private travel with business travel, to set off the entire trip as a business trip against tax liability? | |  | | .868 | |  | |  |
| **Tax avoidance** |  | |  | |  | | .78 | |  |
|  | How likely do you deal with the tax law in detail only to seek opportunities for savings? | |  | | .716 | |  | |  |
|  | How likely would you take a course to learn more about tax saving possibilities? | |  | | .717 | |  | |  |
|  | How likely would you perform a housing refurbishment, simply because this would affect tax reduction? | |  | | .845 | |  | |  |
|  | How likely would you participate in further education, just to use training tax allowances that reduce your tax burden? | |  | | .823 | |  | |  |
| **Proactive tax behavior** |  | |  | |  | | .94 | |  |
|  | Upon request, I would completely deliver all my tax records to the tax authority. | | .881 | |  | |  | |  |
|  | If I fill in my tax return, I strictly stick to the fill-in information and information brochures of the tax office. | | .855 | |  | |  | |  |
|  | Upon request, I would provide all my documents for the tax authorities on time. | | .949 | |  | |  | |  |
|  | If the tax office sets deadlines or appointments, I would adhere to them. | | .927 | |  | |  | |  |
|  | I act cooperatively towards the tax authority. | | .872 | |  | |  | |  |
| **Stall tax behavior** |  | |  | |  | | .82 | |  |
|  | Upon request, I would try to pass only the most necessary documents to the tax authority. | |  | | .824 | |  | |  |
|  | I try to be difficult to reach for the tax authority. | |  | | .640 | |  | |  |
|  | If there are ways to delay deadlines, which are set by the tax authority, I would use them. | |  | | .833 | |  | |  |
|  | Upon request, I only give that information to the tax authority which I am asked for but not more. | |  | | .693 | |  | |  |
|  | If the tax authority wants to get information from me, I would wait with my answer, as long as I can. | |  | | .814 | |  | |  |

*Note:* Items are translations from the original German items.

**Table A3**. Correlations between scales

|  | M (SD) | CP | RP | LP | IT | RBT | TH | TE | TA | ATB | STB |
| --- | --- | --- | --- | --- | --- | --- | --- | --- | --- | --- | --- |
| Coercive Power (CP) | 5.04 (1.39) | - | -.40*** | -.29** | -.30** | -.25* | .07 | .00 | .10 | .05 | .12 |
| Reward Power (RP) | 3.35 (1.49) |  | - | .28** | .36*** | .37*** | -.15 | -.09 | .06 | -.16 | -.09 |
| Legitimate Power (LP) | 3.57 (1.03) |  |  | - | .67*** | .79*** | .17 | -.21* | .13 | .21* | -.26* |
| Implicit Trust (IT) | 3.49 (1.50) |  |  |  | - | .85*** | .19 ^t^ | -.19 ^t^ | .09 | .20 ^t^ | -.29** |
| Reason based trust (RBT) | 3.23 (1.49) |  |  |  |  | - | .13 | -.20 ^t^ | .17 ^t^ | .10 | -.19 ^t^ |
| Tax Honesty (TH) | 6.23 (1.11) |  |  |  |  |  | - | -.49*** | .17 ^t^ | .76*** | -.40*** |
| Tax Evasion (TE) | 2.83 (1.72) |  |  |  |  |  |  | - | -.044 | -.36*** | .49*** |
| Tax Avoidance (TA) | 3.71 (1.49) |  |  |  |  |  |  |  | - | .09 | .15 |
| Accepting tax behavior (ATB) | 6.11 (1.12) |  |  |  |  |  |  |  |  | - | -.41*** |
| Stall tax behavior (STB) | 3.56 (1.46) |  |  |  |  |  |  |  |  |  | - |

*Note*: Partial correlations (controlling for gender, age and turnover). *, **, *** represent statistical significance at the *p* < .05, *p* < .01, *p* < .001 levels, respectively. ^t^ represents a p-value < .10
